# Mutations in ammonia transporter RhBG that impair NH_3_/NH_4_^+^ transport in patients with chronic kidney disease

**DOI:** 10.1113/JP288958

**Published:** 2026-02-11

**Authors:** He Zhou, Solange Abdulnour‐Nakhoul, L. Lee Hamm, Nazih L. Nakhoul

**Affiliations:** ^1^ Department of Medicine Tulane Medical School New Orleans LA USA; ^2^ Department of Physiology Tulane Medical School New Orleans LA USA

**Keywords:** ammonia, ammonium, chronic kidney disease, CKD, electrophysiology, Rh protein, RhBG

## Abstract

**Abstract:**

Chronic kidney disease (CKD) imposes a substantial health burden globally, with emerging evidence pointing to the significance of metabolic acidosis and low urinary NH_4_^+^ excretion resulting in poor CKD outcomes. The present study aims to identify in CKD patients, loss of function mutations in RhBG, one of the NH_3_/NH_4_^+^ transporters in the collecting duct, and to show that NH_3_/NH_4_^+^ transport is impaired by these mutations. Single nucleotide polymorphisms of RhBG associated with CKD occurrence were identified using ancestry‐stratified data from the Chronic Renal Insufficiency Cohort (CRIC) study. Functional analysis of NH_3_/NH_4_^+^ transport was conducted in *Xenopus* oocytes expressing RhBG protein or mutants. NH_3_ and NH_4_^+^ transport was evaluated by electrophysiological measurements, including whole cell current, surface pH and intracellular pH. Our study identified six critical RhBG mutations associated with CKD. G86S and G86C inhibited the transport of NH_3_; mutations G148R and G148W completely blocked transport of NH_3_ and NH_4_^+^, whereas T250A and T250S only inhibited NH_3_ transport. Mutation T250M completely inhibited transport of both NH_3_ and NH_4_^+^. Our study identified critical rare non‐synonymous single nucleotide polymorphisms in RhBG associated with CKD and elucidated the impact of these variants on NH_3_/NH_4_^+^ transport. These data are crucial to our understanding of how mutations can disrupt NH_3_/NH_4_^+^ transport, potentially affecting kidney function in CKD patients susceptible to acidosis.

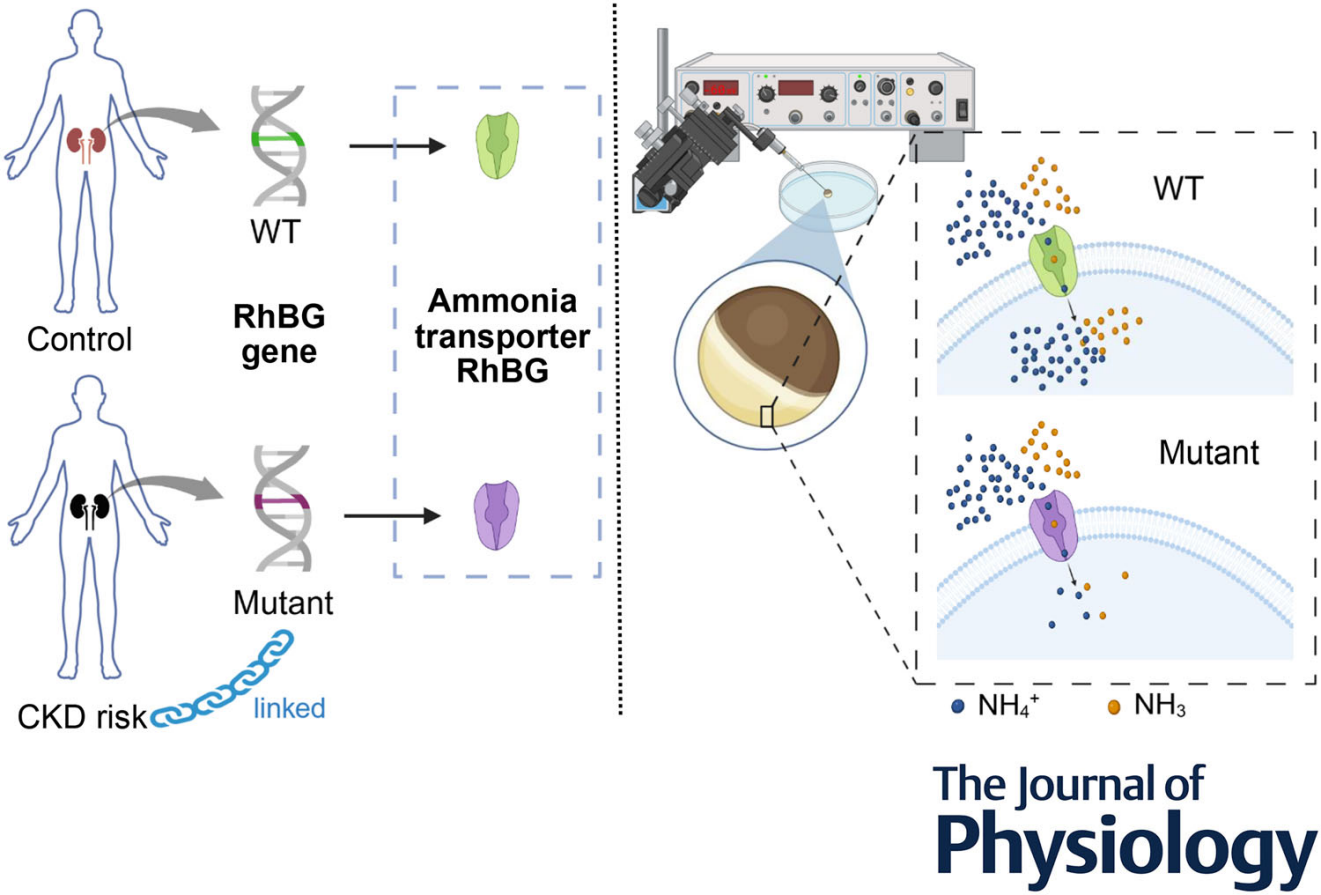

**Key points:**

- Acidosis and low urinary ammonium excretion contribute to poor outcomes in chronic kidney disease (CKD).
- This study investigates how the function of an ammonia transporter in renal collecting duct (RhBG) may contribute to CKD.
- Here, we report six rare RhBG mutations associated with CKD, identified using data from the Chronic Renal Insufficiency Cohort (CRIC) study.
- Using electrophysiological measurements, functional analysis in *Xenopus* oocytes showed that these RhBG mutations disrupt ammonia transport, with some mutations affecting only NH_3_ transport, whereas others affect both NH_3_ and NH_4_^+^ transport.
- The results suggest that impaired ammonia transport by RhBG contributes to CKD, highlighting the need to understand mechanisms that link function (NH_3_/NH_4_^+^ and acid‐base regulation) and genetic predisposition to CKD.

## Graphical abstract

**Abstract figure legend** Overview of the study design and workflow. Left: screening for RhBG mutations associated with chronic kidney disease. Right: functional validation of identified mutations using *Xenopus* oocyte electrophysiology with two‐electrode voltage clamp technique and intracellular pH measurements, showing impairment of NH_3_/NH_4_^+^ transport.

## Introduction

Chronic kidney disease (CKD) involves a loss of renal function over time (Maringhini & Zoccali, 2024). As CKD progresses, the ability of the kidneys to excrete the daily load of acids, predominately generated from metabolism, is impaired (Nagami & Hamm, 2017; Nagami & Kraut, 2022). This leads to the retention of hydrogen ions (H^+^) and eventually an associated decline in plasma pH, namely metabolic acidosis.

The kidneys respond to acidosis by increasing urinary excretion of total ammonia (NH_3_ and NH_4_^+^) and titratable acids. The excretory capacity for ammonia (50–67%) accounts for the major urinary acid excretion compared to titratable acids (33–50%) (Hamm et al., 2015). In the human kidney, total ammonia is generated predominantly in the proximal tubule by deamination of glutamine and secreted into the lumen, reabsorbed in the thick ascending limb of Henle's loop, and then secreted into the collecting duct. In the collecting duct, NH_3_/NH_4_^+^ is transported by two NH_3_/NH_4_^+^ transporters, RhBG and RhCG (Han et al., 2013; Quentin et al., 2003). RhBG, that is, Rhesus (Rh) glycoprotein type B, transports both forms of ammonia (NH_3_ and NH_4_^+^) (Nakhoul & Lee Hamm, 2013; Nakhoul et al., 2005, 2006; Nakhoul, Abdulnour‐Nakhoul, Boulpaep, et al., 2010; Nakhoul, Abdulnour‐Nakhoul, Schmidt, et al., 2010) at the basolateral membrane of α‐intercalated cells and non‐A, non‐B cells (Han et al., 2013).

In the early stage of CKD, H^+^ can accumulate in such organs as kidneys and bones, even without affecting serum bicarbonate levels; this has been referred to as eubicarbonatemic metabolic acidosis (Nagami & Kraut, 2022). As CKD progresses, the increase in ammonium excretion in response to acidosis is impaired, and therefore the prevalence of overt metabolic acidosis increases (Kim, 2021). Recent studies suggest that the reduced ability of the kidney to excrete ammonium is associated with detrimental outcomes in CKD (such as death and cardiovascular events) and faster progression towards end‐stage renal disease (ESRD), measured as a decline in glomerular filtration rate (GFR) over time (Kim, 2021; Raphael et al., 2017; Rehman et al., 2023; Vallet et al., 2015; Wesson et al., 2020). This association between lower urinary total ammonia excretion and CKD progression (ESRD or faster GFR decline) has been found even without overt metabolic acidosis (Madias, 2021; Tyson et al., 2021; Vallet et al., 2015) independently of measured GFR and other factors (Madias, 2021). These findings suggest that lower urinary ammonium excretion can serve as an early indicator of higher CKD risk, even earlier than serum total CO_2_ level (Raphael et al., 2017). Lower ammonium excretion and more retention of acid may be an important pathogenic factor, not just a marker, in CKD progression as well.

Ammoniagenesis is enhanced to increase bicarbonate production to counteract acidosis (DuBose, 2017; Madias, 2021; Wesson et al., 2020). The enhanced ammoniagenesis probably also exists in early‐stage CKD during preclinical acidosis given that dietary acid load, in those patients, increased ammonia production without discernible systemic acidosis (DuBose, 2017). Tissue‐specific knockout studies in mice have reported a correlation between Rhbg (homologue of RhBG) deletion and exacerbated metabolic acidosis with acid challenge (Bishop et al., 2010; Lee et al., 2014). A higher risk of CKD progression has been observed in human subjects with early‐stage CKD subjected to a high‐protein diet as acid loading. Acid loading was determined by measurement of urinary net acid excretion (Banerjee et al., 2015; Scialla et al., 2012, 2017). Those important findings suggest a critical role of ammonia excretion, possibly mediated by RhBG.

Because current evidence supporting the link between low urinary ammonium excretion and poor CKD outcomes is heavily based on observational studies, there is a critical need to provide biomedical evidence to validate the importance of clinically measuring urinary ammonium in patients. In the present study, our hypothesis is that impaired transport of NH_3_/NH_4_^+^ by RhBG is a factor that contributes to CKD. Although other processes and transporters can affect ammonium excretion, the focus here is on RhBG.

The aims of the present study are to: (i) identify human RhBG non‐synonymous single nucleotide polymorphisms (nsSNPs) that are possibly linked to CKD and (ii) validate the functional impacts of such SNPs on total ammonia transport. To do so, we first conducted association analyses using whole‐exome sequencing (WES) data collected from participants in the Chronic Renal Insufficiency Cohort (CRIC) study and identified SNPs with significant associations with CKD outcome. We then performed functional studies to investigate the biological significance of the identified SNPs using electrophysiological techniques. Our results show that: (i) six mutations (G86S, G86C, G148R, G148W, T250A and T250S) are associated with CKD in the study cohort; (ii) two mutations associated with CKD progression, G86S and G86C, partially inhibited transport, with G86C being more deleterious than G86S in its inhibitory effect on NH_3_/NH_4_^+^ transport; (iii) mutations G148R, G148W and T250M completely inhibited the transport of NH_3_ and NH_4_^+^, demonstrating that they are critical sites for RhBG protein function.

## Methods

### Ethical approval

The protocols for housing and handling of *Xenopus laevis* frogs and the experimental protocols were reviewed and approved by the Institutional Animal Care and Use Committee at Tulane University (approval ID number 1802).

### Animal care

Frogs were housed in a 100 gallon water tank, typically half‐filled, that held a minimum of six and up to a maximum of 20 frogs at a time. The tank water was dechlorinated and circulated through a charcoal‐filtered power pump. The size of the tank and volume of water allowed frogs to swim freely. The tank was supplied with plastic pipes for activity enrichment. The frogs were fed three times per week with adult *Xenopus* diet (Nasco Frog Brittle; Nasco Education, Fort Atkinson, WI, USA) sprinkled directly into the tank. After each feeding, the frogs were removed to a spare similar tank and the first tank was cleaned and refilled with water.

### Animal procedures

To extract oocytes, *Xenopus* frogs were anaesthetized via the transdermal route by immersion in filtered water containing 0.2% tricaine (ethyl 3‐aminobenzoate methanesulfonate; Sigma, St Louis, MO, USA) for 15 min. Adequate anaesthetic depth was confirmed by the absence of withdrawal reflexes upon application of mechanical stimulus to the hindlimb. Oocytes were then extracted as described below (see section below, ‘Oocyte isolation’). Following oocyte removal, the frogs were monitored for 30–60 min to ensure complete recovery from anaesthesia before being transferred to individual recovery bins overnight without feeding. Frogs were then returned to standard housing conditions. Our protocol allows six survival surgeries, after which the frog was killed by deep anaesthesia (immersion in 0.2% tricaine for 2 h) followed by cardiac excision.

### Sex as a biological variable

Our studies on association tests in humans included data on both males and females. These data obtained from public databases included both sexes, but sex was not considered a biological variable as a result of an insufficient sample size for stratification by sex. Only female *Xenopus* frogs were used in this study to extract oocytes.

### Identification of RhBG nsSNPs associated with CKD

#### Study population

We obtained the WES dataset of the CKD patients in CRIC study and selected 507 participants of African ancestry and 1147 of European ancestry. All selected cases had eGFR <60 mL min^−1^ per 1.73 m^2^ or spot urine albumin‐to‐creatinine ratio ≥30 µg mg^−1^ (Pan et al., 2023). Cases were compared with healthy controls selected from the Atherosclerosis Risk in Communities (ARIC) study that included 6044 subjects of European ancestry and 2056 of African ancestry. All selected healthy controls had eGFR ≥90 mL min^−1^ per 1.73 m^2^.

#### Association analysis

Ancestry‐stratified nsSNP association tests were performed using RStudio (R version 4.3.1; R Foundation, Vienna, Austria). nsSNPs of RhBG present in the CRIC WES dataset with a minor allele frequency (MAF) ≤ 1% were tested individually for association between cases and controls. Rare variants (MAF ≤ 1%) are prioritized because they more probably have larger effect sizes on the function. MAF values were calculated using standard allele frequency methodology ($\mathrm{MAF}\;=\frac{\mathrm{copies}\;\mathrm{of}\;\mathrm{the}\;\mathrm{alternate}\;\mathrm{allele}\;}{\mathrm{total}\;\mathrm{copies}\;\mathrm{of}\;\mathrm{all}\;\mathrm{alleles}}\;$), computed separately for each ancestry group in CRIC and ARIC cohorts. Pearson's chi‐squared test with Yates' continuity correction was chosen for simplicity and its non‐parametric nature. The odds ratio (OR) was also calculated to measure the effect size using Fisher's exact test given our focus on rare variants. For certain nsSNPs with MAF of 0 in our sample, risk difference (RD) instead of OR was reported along with the *P* value of Fisher's exact test. All *P* values were adjusted with the Bonferroni correction method by pooling the two ancestry groups to reach maximum robustness. *P* < 0.05 was considered statistically significant. Findings with significant OR or RD after correction (*P* < 0.05) were used for functional testing. This study did not examine common variants (MAF > 1%).

#### Selection of potentially critical nsSNPs

The number of RhBG nsSNPs in our study population that met selection criteria was small (*N* = 10) (Table 1) and only four reached statistical significance (Table 2). Among these four nsSNPs, rs150963900 (F81L) in the European group showed a protective effect (OR < 1) which is outside our study scope. Therefore, we excluded it from the functional test. nsSNP rs200320178 (T250M) causes a missense mutation of the same amino acid as rs760016272 (T250A/S). To explore the potential effect of this critical amino acid, we included rs200320178 (T250M) in downstream functional analyses.

**Table 1:** List of rare single nucleotide variants (SNVs) from the CRIC study

		African ancestry (*N* = 2563)		European ancestry (*N* = 7191)	
Variant.ID	Amino acid substitution	Case MAF (*N* = 507)	Control MAF (*N* = 2056)	Case MAF (*N* = 1147)	Control MAF (*N* = 6044)
rs202003473	Y36C	0	0	0.001689	0.000437
rs370957772	L82R	0	0	0.001689	0.000146
rs200069134	G86S/C	0	0	0.005068	0.001091
rs575652633	G148R/W	0	0	0.001689	0
rs190623502	R211C	0	0	0.001689	0.000509
rs760016272	T250A/S	0	0	0.001689	0
rs200320178	T250M	0	0.000632	0.003378	0.003420
rs201484566	A286G	0.001684	0.000632	0	0
rs376569860	L358P	0.001684	0.001476	0	0
rs150963900	F81L	0.003367	0.001896	0.013510	0.022700

Selected SNVs from the WES data of the subgroup of CRIC study with MAF falling in the interval (0, 1%]. Only 10 SNVs meet the criteria. Controls are from the ARIC WES dataset. *N* indicates the sample size of participants in each subgroup based on disease and ancestry. Allele counts are not shown. ARIC, Atherosclerosis Risk in Communities; CRIC, Chronic Renal Insufficiency Cohort; MAF, minor allele frequency; WES, whole‐exome sequencing.

**Table 2:** Results of ancestry‐stratified single‐variant association tests

Variant.ID	Amino acid substitution	Ancestry	Chi‐squared	Chi‐square *P* value	OR	RD	Fisher's exact *P* value	*P* value corrected	*P* value significance
rs200320178	T250M	African	0.018353	0.8922		−0.1979309	1	1	
rs201484566	A286G	African	0.32965	0.5659	2.706786		0.2581265	1	
rs376569860	L358P	African	1.00E‐27	1	1.158403		0.6947681	1	
rs150963900	F81L	African	8.91E‐03	0.9248	1.355423		0.7145403	1	
rs202003473	Y36C	European	3.5427	0.05981	4.225876		0.04139785	0.4553763	
rs370957772	L82R	European	8.0594	0.004527	10.56771		0.00734661	0.08081271	
rs200069134	G86S/C	European	16.967	3.80E‐05	4.902102		0.0001724	0.001896389	**
rs575652633	G148R/W	European	15.307	9.14E‐05		0.8409628	0.00064208	0.007062902	**
rs190623502	R211C	European	2.7158	0.09936	3.521293		0.06049482	0.665443	
rs760016272	T250A/S	European	15.307	9.14E‐05		0.8409628	0.00064208	0.007062902	**
rs200320178	T250M	European	1.69E‐26	1	1.027837		0.8471217	1	
rs150963900	F81L	European	7.6372	0.005718	0.5849269		0.00346187	0.0380806	*

Chi‐squared was calculated with Pearson's chi‐squared test with Yates' continuity correction, degree of freedom = 1. The odds ratio was calculated with Fisher's exact test. Allele counts are not shown. Risk difference (RD) was calculated for variants with one MAF of 0 with Fisher's exact *P* value reported. Fisher's exact *P* values are adjusted to account for multiple comparisons with Bonferroni method. Corrected *P* < 0.05 is considered statistically significant. OR, odds ratio; RD, risk difference.

### Functional tests of selected RhBG nsSNPs

Functional testing of RhBG mutations was conducted by expressing RhBG mutants in *Xenopus* oocytes and measuring NH_3_/NH_4_^+^ transport. NH_3_/NH_4_^+^ transport was assessed by measurements of NH_3_/NH_4_^+^‐induced currents, intracellular pH (pH_i_) and surface pH (pH_s_), as described below.

#### Site‐directed mutagenesis

Human RhBG clone was purchased from GeneScript and subcloned into pGH19 plasmid and purified by QIAprep Miniprep kit (Qiagen, Santa Clarita, CA, USA).

##### Mutagenic primer design

Complementary (100% overlap) mutagenic oligonucleotide primers were designed individually according to the desired mutation (Table 3). Each pair of mutagenic primers was between 25 and 45 bases in length with a high annealing temperature (≥78°C) and a minimum GC content of 40% and terminated in one or more C or G bases. The desired mutation was flanked by at least 10 bases of unmodified sequence on both sides. To increase the success rate of getting the desired mutations, we conducted two‐stage PCR amplification (Wang & Malcolm, 2002). Purified forward and reverse primers were added to two thin‐walled PCR tubes separately. After adding the circular plasmid DNA template and other PCR reagents (buffer, dNTPs, ddH_2_O, dimethyl sulfoxide, PfuTurbo polymerase) (#600600; Agilent, Santa Clara, CA, USA) to each tube (50 µL reaction volume), we performed PCR following a regular three‐step PCR protocol. After five cycles at the annealing stage, we paused the program, took a half‐volume of PCR mix (25 µL) from each tube containing forward or reverse primer to combine in a new tube and allow the combined tube to continue PCR for another 16 cycles followed by the extension and cooling stages.

**Table 3:** List of SNPs included in functional tests and primers used for mutagenesis

Variant.ID	Amino acid substitution	Nucleotide substitution	Primers for SDM
rs200069134	G86S	G>A	[R] 5′‐cacgctgctgaagctgtaacgctgcagga‐3′ [F]5′‐tcctgcagcgttacagcttcagcagcgtg‐3′
rs200069135	G86C	G>T	[R] 5′‐cacgctgctgaagcagtaacgctgcagga‐3′ [F] 5′‐tcctgcagcgttactgcttcagcagcgtg‐3′
rs575652633	G148R	G>C	[R] 5′‐gctgggtaggccgggtcttgcccag‐3′ [F] 5′‐ctgggcaagacccggcctacccagc‐3′
rs575652634	G148W	G>T	[R] 5′‐gctgggtaggccaggtcttgcccag‐3′ [F] 5′‐ctgggcaagacctggcctacccagc‐3′
rs760016272	T250A	A>G	[R] 5′‐tgttgagggccgcccgatgctgccc‐3′ [F]5′‐gggcagcatcgggcggccctcaaca‐3′
rs760016273	T250S	A>T	[R] 5′‐tgttgagggccgaccgatgctgccc‐3′ [F] 5′‐gggcagcatcggtcggccctcaaca‐3′
rs200320179	T250M	C>T	[R] 5′‐ttgagggccatccgatgctgcccagcc‐3′ [F] 5′‐ggctgggcagcatcggatggccctcaa‐3′

Abbreviations: F, forward; R, reverse; SDM, site‐directed mutagenesis.

##### Transformation

PCR product was treated with *Dpn*I restriction enzyme (New England Biolabs, Ipswich, MA, USA) and incubated at 37°C for 1 h to digest the template DNA. *Dpn*I treated PCR product was then transformed into XL10 gold Ultracompetent cells (#200517‐4; Agilent) for amplification, antibiotic selection, plasmid isolation and purification. We purified plasmid DNA from selected colonies and confirmed the mutation by Sanger sequencing (GENEWIZ; Azenta Life Science, Burlington, MA, USA).

#### cRNA preparation

The purified pGH19 plasmid containing RhBG cDNA [wild‐type (WT) or with mutations] was digested with *Nhe*1 enzyme (New England Biolabs) to create a linear template, followed by digestion with proteinase K. The DNA was then extracted using phenol‐chloroform extraction and ethanol precipitation. Capped RNA (cRNA) transcripts were synthesized *in vitro* using the mMachine T7 transcription kit (Ambion, Austin, TX, USA). The concentration of cRNA was measured using a Nanodrop spectrophotometer (Thermo Fisher Scientific, Waltham, MA, USA) and its quality was evaluated using formaldehyde/Mops/1% agarose gel electrophoresis.

#### Oocyte isolation

All experiments were conducted on *X. laevis* oocytes. Isolation of oocytes followed standard protocols as reported in previous study (Nakhoul et al., 2005). Briefly, the frog was anesthetized by immersion in water containing 0.2% tricaine. A 1 cm incision was made in the abdominal wall to access a portion of the ovary, which was then cut. The wound was closed with stitches in the muscular layer using 5‐0 chromic gut and, in the skin, using 6‐0 silk. The excised ovary piece containing oocytes was rinsed with Ca^2+^‐free ND96 solution, then agitated in Ca^2+^‐free ND96 solution containing collagenase type IV (0.3 mg mL^−1^). After 30–40 min, free oocytes were rinsed with sterile OR3 medium, pH 7.5 and osmolality of 200 mOsm kg^−1^ L‐15 medium [(L‐4386; Sigma), 5x penicillin‐streptomycin (P0781; Sigma) and 5.5 mM Hepes (H4034; Sigma)], and then sorted and stored at 18°C overnight before injection.

#### Oocyte injection

Each oocyte from the same batch was injected with 50 nL of WT‐RhBG or mutant cRNA (0.2 µg µL^−1^, totalling 10 ng of RNA) for protein expression or double‐distilled H_2_O (Thermo Fisher Scientific). Water‐injected oocytes served as controls to account for endogenous NH_4_^+^‐permeable channel activity. The injections were made using sterile pipettes with tip diameters of 20–30 µm, connected to a Nanoject motor‐driven pipette (Drummond Scientific, Broomall, PA, USA). Injected oocytes were observed 3 days post‐cRNA injection and used for the following 4–5 days. Injection for all experimental and control groups was repeated in three or more batches of oocytes.

#### Electrophysiological measurements

All measurements were performed on oocytes expressing WT‐RhBG or mutants. Oocytes were placed in a perfusion chamber (Warner Instruments, Holliston, MA, USA) designed to allow continuous fast perfusion of different solutions. Perfusion rate was 10 mL min^−1^ and complete change of solutions in the chamber was accomplished in 6 s. Voltage clamp experiments, surface pH (pH_s_) and intracellular pH (pH_i_) measurements were extensively described previously (Geyer et al., 2013; Nakhoul et al., 2005, 2006). Measurements on each group were repeated in at least three batches of oocytes, with similar group sizes maintained across batches to ensure balanced representation.

#### Perfusion solutions

Control solution (referred to as ‘Hepes’ in this paper) was made from ND96 medium containing 96 mM NaCl, 2 mM KCl, 1 mM MgCl_2_ and 1.8 mM CaCl_2_ and buffered with 5 mM Hepes to pH 7.5. Two types of testing solution were prepared: (i) NH_3_/NH_4_^+^ solution was prepared by replacing NaCl with NH_4_Cl resulting in 5 mM NH_4_Cl and (ii) methylamine/methylammonium (MA/MA^+^) solution was made by replacing NaCl with methylamine hydrochloride resulting in 5 mM MA/MA^+^. All solutions had osmolarities of 200 ± 5 mOsm L^−1^. Unless otherwise noted, all chemicals and reagents used in this study were purchased from Sigma‐Aldrich (St Louis, MO, USA).

#### Two‐electrode voltage clamp

Whole‐cell currents were measured using a two‐electrode voltage clamp system (OC‐725; Warner Instruments). Borosilicate glass capillaries (1.5 mm outer diameter; Warner Instruments) were pulled using a horizontal puller (model P‐97; Sutter Instruments, Novato, CA, USA) to create electrodes with resistances of 1–4 MΩ, filled with 3 M KCl solution. The external reference electrodes were a long glass micropipette with a tip of ∼10 µm filled with 3 M KCl. A Ag/AgCl wire, dipped in the 3 M KCl filling solution provided electrical connection to the electrometer. It was directly immersed in the chamber. Oocytes were clamped at −60 mV, and continuous current readings were sampled at a rate of once per second. Inward flow of cations was detected as inward current (negative current).

#### Measurement of surface pH (pH_s_)

Single‐barreled alumino‐silicate glass capillaries (1.5 mm outer diameter, 0.86 mm inner diameter; Frederick Haer, Brunswick, MD, USA) were pulled to form microelectrodes with a tip of ∼12 µm, then dried in an oven at 200°C for 2 h. They were then silanized with bis(dimethylamino)‐dimethyl silane vapor in a closed vessel (300 mL). The tip of each microelectrode was filled with H^+^ ionophore cocktail B (#95293; Fluka Chemical Corp., Ronkonkoma, NY, USA) to form a pH microelectrode. The remaining space of the microelectrode was backfilled with a buffer solution (Ammann et al., 1981; Nakhoul, Abdulnour‐Nakhoul, Boulpaep, et al., 2010). The electrode signal was measured with a FD223 electrometer (World Precision Instruments, Sarasota, FL, USA). The external reference electrode was prepared in the same way as for the two‐electrode voltage clamp. During the experiment, the pH_s_ electrode was placed in direct contact with the oocyte surface after the calibration in bath. The electrode in‐position does not impale the oocyte but should cause a visible slight dimple on the oocyte membrane.

#### Measurement of intracellular pH (pH_i_)

Microelectrodes for measuring pH_i_ were prepared in a similar way to pH_s_ microelectrode except that they were pulled to a smaller tip (<0.2 µm). Ling–Gerard (voltage) microelectrodes were pulled from 1.5 mm (outer diameter) borosilicate glass capillaries with an inner filament and filled with 3 m KCl. An Ag/AgCl wire was inserted in the 3 M KCl filling solution. The external reference electrode was prepared in the same way as above. During the experiment, the oocyte was impaled with both a pH_i_ electrode and a voltage electrode for sensing pH and membrane potential simultaneously. The outputs of the FD223 electrometer (both *V*_m_ and pH) were fed to an electronic subtraction box (Yale, New Haven, CT, USA) to obtain net pH_i_ voltage. Electrical outputs were digitized and custom‐made software displayed net pH and *V*_m_ readings online.

#### Immunofluorescence to test expression

The preparation was described in detail previously (Abdulnour‐Nakhoul et al., 2016). Briefly, oocytes injected with cRNA (WT or mutant) or water (as the control) were snap‐frozen in OCT compound over dry ice. Cryo‐sections (5 µm) were fixed, rehydrated, and incubated with human RhBG antibody (dilution 1:500; #AAS03495C; Antibody Verify, Las Vegas, NV, USA), followed by goat anti‐rabbit Alexa Fluor 488 secondary antibodies (dilution 1:500; #A‐11008; Invitrogen, Waltham, MA, USA). Negative controls were included. Micrographs were captured using an Eclipse 80i microscope (Nikon, Tokyo, Japan) with a SPOT RT digital camera (SPOT Imaging, Sterling Heights, MI, USA). All immunofluorescence micrographs were captured under identical settings using standardized exposure and acquisition parameters.

### Statistical analysis

All variables in electrophysiological measurements were compared using mixed‐effects models to account for oocyte clustering within injected batches. Outliers were identified using ‘above Q3 + 1.5 × interquartile range (IQR) or below Q1 − 1.5 × IQR’ method and removed prior to all analyses. To assess the consistency of electrophysiological measurements across oocyte batches, intraclass correlation coefficient (ICC) scores were calculated using mixed‐effects models. For each parameter, a null model (including only the random effect of batch) and a full model (including both fixed and random effects) were fitted using lmer() function in package ‘lme4’ (Bates et al., 2015). ICC values were computed in R using the performance::icc() function in package ‘performance’ (Lüdecke et al., 2021), which estimates adjusted ICC based on experimental oocyte groups (WT, mutants, water‐injected control). Adjusted ICC values reflect the proportion of total variance attributable to batch effects after accounting for fixed effects. ICC scores were interpreted as follows: values <0.2 indicate minimal batch‐related variance, whereas values between 0.2 and 0.5 suggest moderate batch‐related variance. Higher ICC values indicate greater inter‐batch variability, supporting the need for mixed‐effects modelling.

Linear mixed‐effects models were fitted with experimental oocyte groups (WT, mutants, water‐injected control) as fixed effects and batch as a random effect using lmer() function in package ‘lme4’. Linearity of the relationship between experimental groups and electrophysiological measurements was assessed using the Ramsey RESET test in package ‘lmtest’ (Zeileis et al., 2002), which indicated no significant deviation from linearity, supporting the use of a linear model. Estimated marginal means (EMMs) were computed using package ‘emmeans’ (DOI:10.32614/CRAN.package.emmeans) and custom contrasts were defined to compare each mutant group and the water‐injected group against the RhBG control. *P* values of EMM contrasts were adjusted for multiple comparisons using the false discovery rate method to control for type I error. Complete mixed‐effects model outputs are provided in the Appendix (Tables A1 and A2) for methodological transparency.

Descriptive statistics (mean ± SD) represent observed (measured) values and were reported for biological interpretation and data transparency. Observed means of different groups were also analysed with one‐way ANOVA followed by appropriate *post hoc* tests; for methodological comparison and transparency, see Appendix Methods and Table A3. All statistical results were generated in RStudio (R version 4.4.3) and Excel (Microsoft Corp., Redmond, WA, USA), unless otherwise noted.

## Results

### CKD‐related RhBG rare variants

Our study population from the CRIC database included a European ancestry group (73%) and an African ancestry group (27%) (Table 1). There were 10 rare variants (MAF < 1%) identified from the CRIC study WES dataset. Six of these variants were only detected in the group of European ancestry. Only two were in the African group and only one was in both ancestry groups. The results of association tests (chi‐squared test) and effect size (OR or RD) based on Fisher's exact test were corrected with different methods (Table 2). After correction, four SNPs reached statistical significance in both the chi‐squared test and Fisher's exact test (*P* < 0.05). Variant rs150963900 (F81L) has an OR less than one (OR = 0.58), which indicates a protective effect on the outcome (presence of CKD) and was not included in functional experiments. The three other variants (rs200069134, rs575652633 and rs760016272) cause six types of amino acid substitution (G86S, G86C, G148R, G148W, T250A and T250S) (Table 3). Variant rs200320178 (T250M) did not reach statistical significance but it causes a missense mutation at the same amino acid as SNP rs760016272 (T250A/S). The identified variants that are significantly associated with CKD and their primer designs for site‐directed mutagenesis (SDM) are listed in Table 3.

### Effects of expressing human RhBG on current, pH_i_ and pH_s_ changes

**Figure 1:**
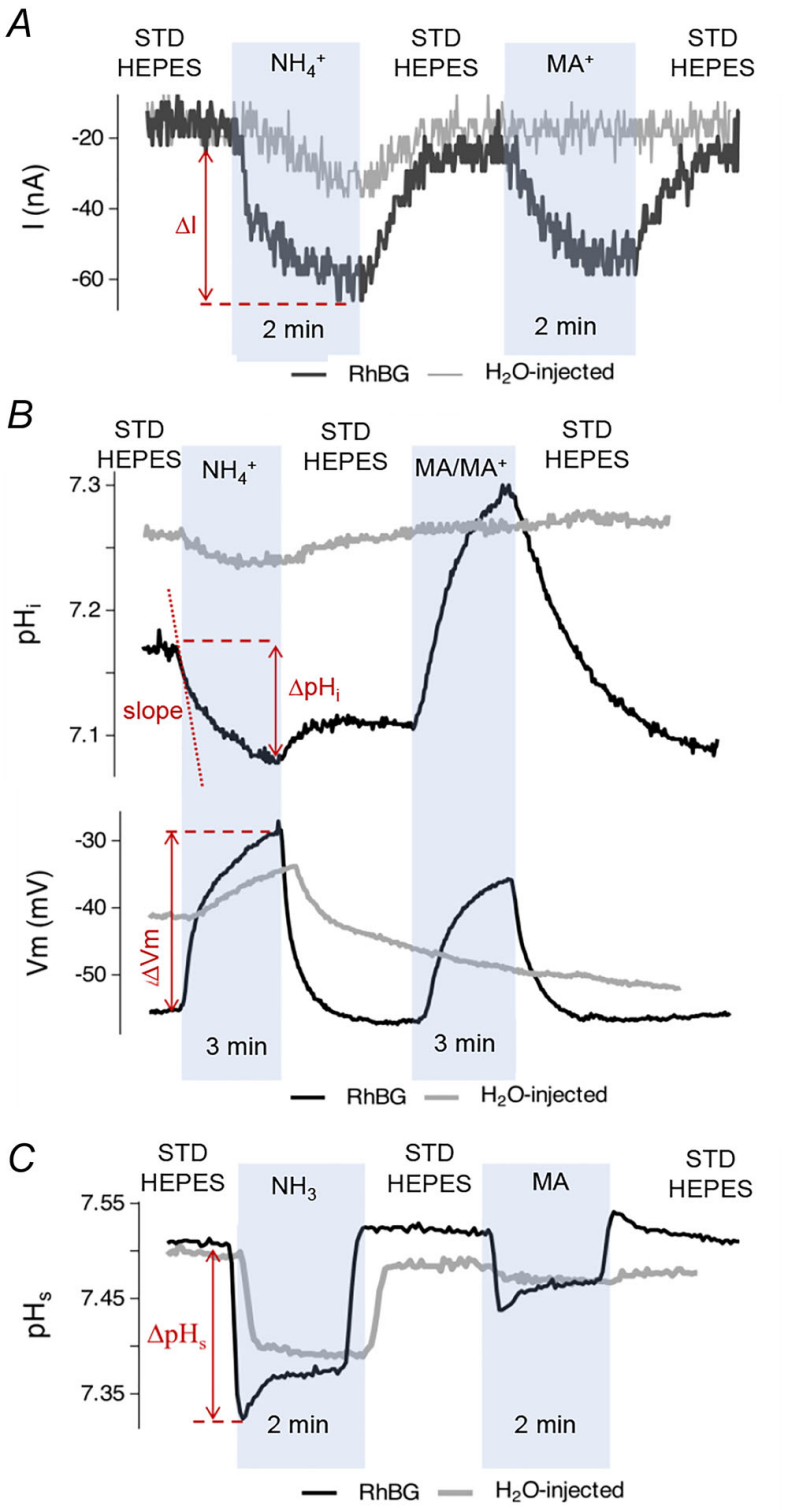
Measurements in oocytes (WT‐RhBG vs. H_2_O) exposed to NH_3_/NH_4_^+^ or MA/MA^+^ A light blue rectangle indicates the period when the oocyte was perfused with testing solutions containing either NH_3_/NH_4_^+^ or MA/MA^+^. STD Hepes represent the time when the oocyte was perfused with control solution (Hepes). Black and grey tracings represent the measurements conducted on the oocyte expressing WT‐RhBG or on the oocyte injected with water as a control, respectively. Each tracing is a single representative experiment. All changes observed were reversed upon removal of test solutions. nA, nanoamp; *V*_m_, membrane potential; mV, millivolt; Δ, measurement difference (end value minus initial value); slope = $\frac{\Delta \mathrm{pHi}}{\Delta \mathrm{time}\;(s)}$× 10^4^. *A*, whole cell transmembrane current (*I*) measurements at a clamped voltage of −60 mV. Positively charged NH_4_^+^ and MA^+^ entry induced inward current in the RhBG‐expressing oocyte. NH_4_^+^ induced a significantly smaller current in the control oocyte than in RhBG‐expressing oocyte (statistics are shown in Fig. 2C). MA^+^ did not cause any current change in the control oocyte. *B*, intracellular pH measurements. In RhBG‐expressing oocyte, the entry of NH_4_^+^ caused a rapid decrease in pH_i_ (bigger absolute value of slope) and a larger depolarization of the membrane potential compared to the control (grey tracing). In RhBG‐expressing oocyte, MA^+^ not only induced an increase in pH_i_ (rather than a decrease) in the RhBG‐expressing oocyte, but also induced depolarization of *V*_m_. There were no significant MA/MA^+^‐induced changes of pH_i_ or *V*_m_ in H_2_O‐injected control oocytes. *C*, surface pH measurements. In RhBG‐expressing oocytes, NH_3_/NH_4_^+^ caused rapid decrease in pHs followed by a partial recovery. Removal of NH_3_/NH_4_^+^ caused full recovery of pHs with a slight overshoot. Exposure to MA/MA^+^ caused a similar pattern of pHs change (rapid decrease followed by partial recovery) but the changes were smaller compared to NH_3_/NH_4_^+^. In H_2_O‐injected oocytes, NH_3_/NH_4_^+^ caused a smaller but sustained decrease in pHs (no partial recovery). MA/MA^+^ did not cause any significant change in pHs. The decrease in pHs is caused by non‐charged NH_3_ and MA entry resulting in a larger ΔpH_s_ in the RhBG‐expressing oocyte than the control (statistics are shown in Fig. 3C and *D*).

In initial experiments, we documented that human RhBG transports NH_3_/NH_4_^+^ and MA/MA^+^ similar to the well‐studied mouse homologues. NH_4_^+^ (or MA^+^) transport will cause an inward current and depolarization of the oocyte membrane, and NH_3_ and MA transport will cause a decrease in pH_s_. Our results show direct measurements of currents and intracellular and surface pH in oocytes expressing the human clone of RhBG exposed to NH_3_/NH_4_^+^ or MA/MA^+^ (Fig. 1). In RhBG‐expressing oocytes, the inward current induced by 5 mm NH_4_^+^ (Fig. 1*A*) was significantly different (−35.7 ± 13.3 nA, *P* < 0.001) compared to H_2_O‐injected control oocytes (−15.6 ± 7.7 nA). MA^+^ at a concentration of 5 mm induced a significant inward current (−27.1 ± 9.88 nA, *P* < 0.001) in RhBG‐expressing oocytes. This inward current was not observed in any H_2_O‐injected control oocytes exposed to MA^+^, which is consistent with previous studies (Ludewig, 2004; Nakhoul et al., 2005; Nakhoul, Abdulnour‐Nakhoul, Boulpaep, et al., 2010). The results of these measurements suggested that the electrogenic transport of NH_4_^+^ and MA^+^ by human RhBG follows a similar pattern to its mouse homologue (Rhbg) as shown in our previous studies (Nakhoul et al., 2005, 2006; Nakhoul, Abdulnour‐Nakhoul, Boulpaep, et al., 2010). Moreover, 5 mm NH_4_^+^ induced a pH_i_ decrease and depolarization in both RhBG‐expressing and H_2_O‐injected oocytes (Fig. 1*B*) as reported previously in studies using mouse Rhbg (Nakhoul et al., 2005, 2006; Nakhoul, Abdulnour‐Nakhoul, Boulpaep, et al., 2010). These changes are consistent with influx of NH_4_^+^. A concentration of 5 mm NH_4_^+^ induced a faster pH_i_ decrease (steeper slope, *P* < 0.001) and larger depolarization of *V*_m_ (*P* < 0.001) in RhBG‐expressing oocytes compared to H_2_O‐injected control oocytes. The effects of MA/MA^+^ on pH_i_ and *V*_m_ caused an increase in pH_i_ (not a decrease) but still caused a depolarization of *V*_m_ (Fig. 1*B*, black tracing) but had no effect on H_2_O‐injected oocytes (Fig. 1*B*, grey tracing). These changes resemble those in oocytes expressing mouse Rhbg (Nakhoul, Abdulnour‐Nakhoul, Boulpaep, et al., 2010).

**Figure 2:**
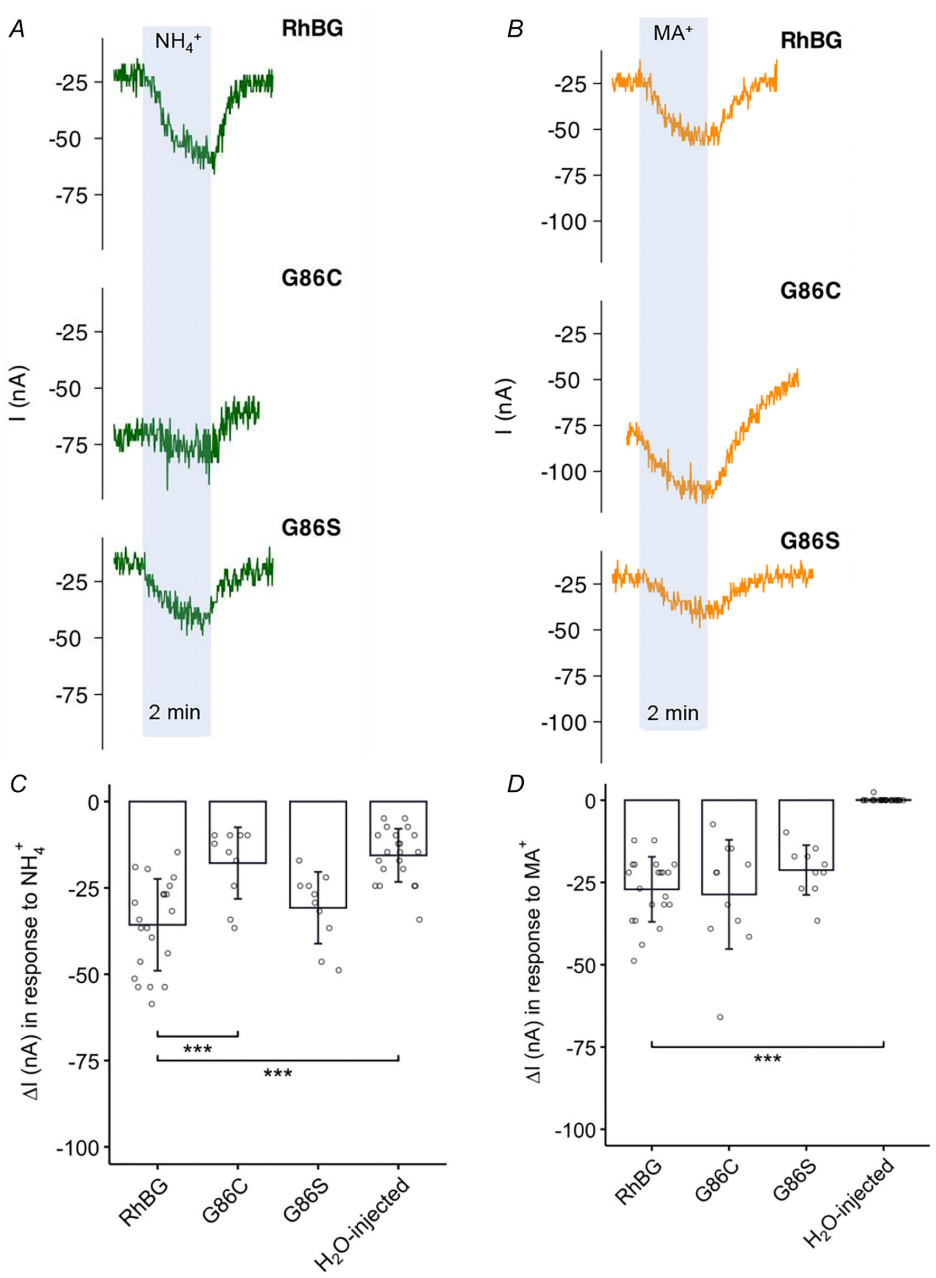
Effects of G86C/S on current induced by NH_4_^+^ and MA^+^ *A*, NH_4_^+^‐induced current (I) in oocytes expressing G86C and G86S mutations compared to WT‐RhBG. *B*, typical tracings of MA^+^‐induced current of oocytes expressing G86C and G86S mutations compared to WT‐RhBG. *C* and *D*, bar plots of current change (Δ*I*) induced by NH_4_^+^ and MA^+^, respectively. Error bars were created using the SD. Individual data points represent individual oocyte measurements pooled from three independent injection batches after outlier removal. RhBG, *N* = 21; G86C, *N* = 11; G86S, *N* = 10; H_2_O‐injected, *N* = 21. ***P < 0.001. Statistical significance was adjusted for multiple comparisons using the false discovery rate method. Bar plots were adapted from multiple comparison tests and broken into separate plots based on mutation groups for clear illustration. For *P* values, see Table 5.

**Figure 3:**
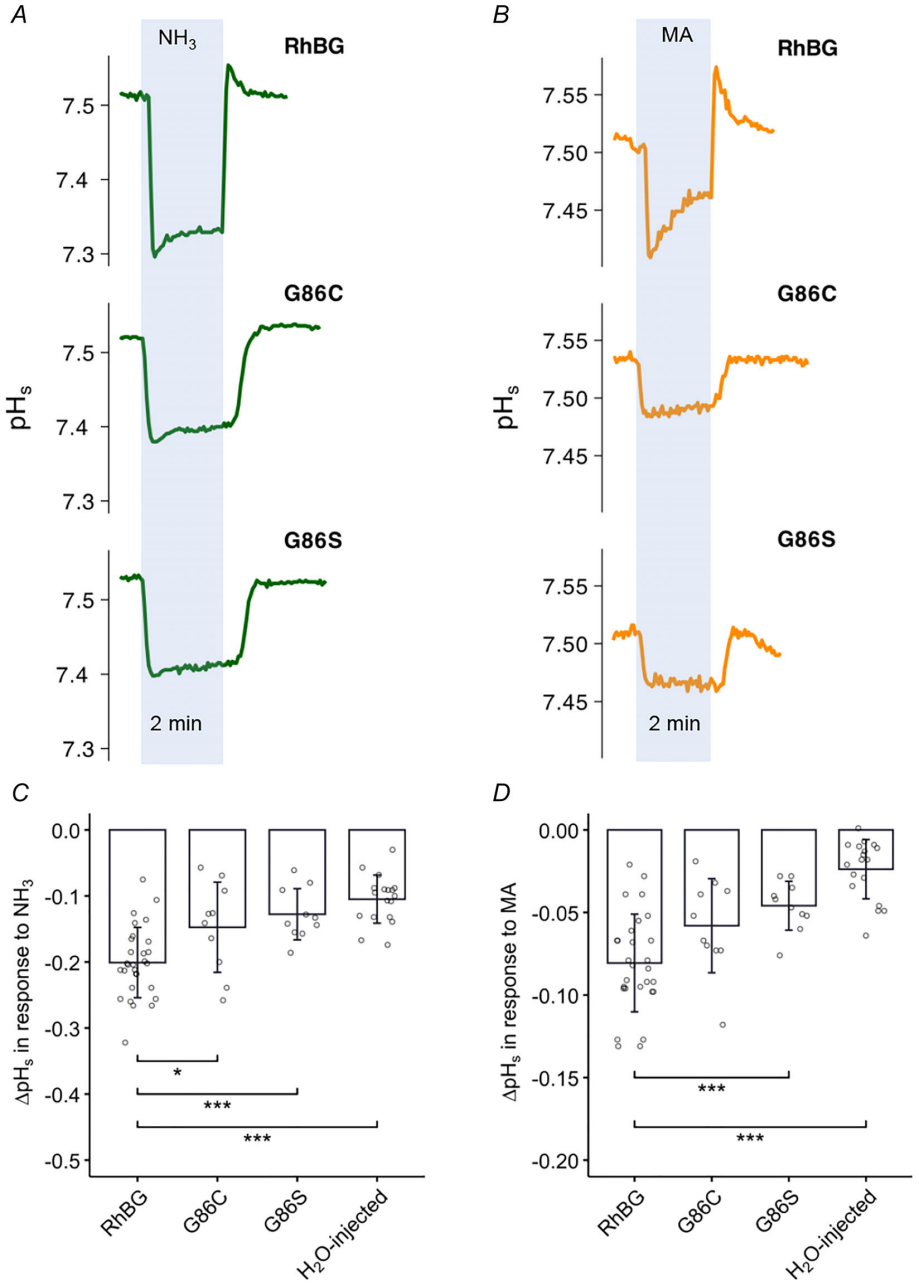
Effects of G86C/S on pH_s_ induced by NH_3_ and MA *A*, tracings showing NH_3_‐induced pH_s_ changes of G86C and G86S mutations compared to WT. *B*, tracings showing MA‐induced pH_s_ changes of G86C and G86S mutations compared to WT. *C* and *D*, summary bar plots of pH_s_ changes (pH units) induced by NH_3_ and MA, respectively. Error bars were created using the SD. Individual data points represent individual oocyte measurements pooled from three independent injection batches after outlier removal. RhBG, *N* = 29; G86C, *N* = 10; G86S, *N* = 10; H_2_O‐injected, *N* = 17. ***P < 0.001; *P < 0.05. Statistical significance of multiple comparisons by mixed‐effects models was corrected using the false discovery rate method. Bar plots were adapted from multiple comparison tests and broken into separate plots based on mutation groups for clear illustration. For *P* values, see Table 5.

**Figure 4:**
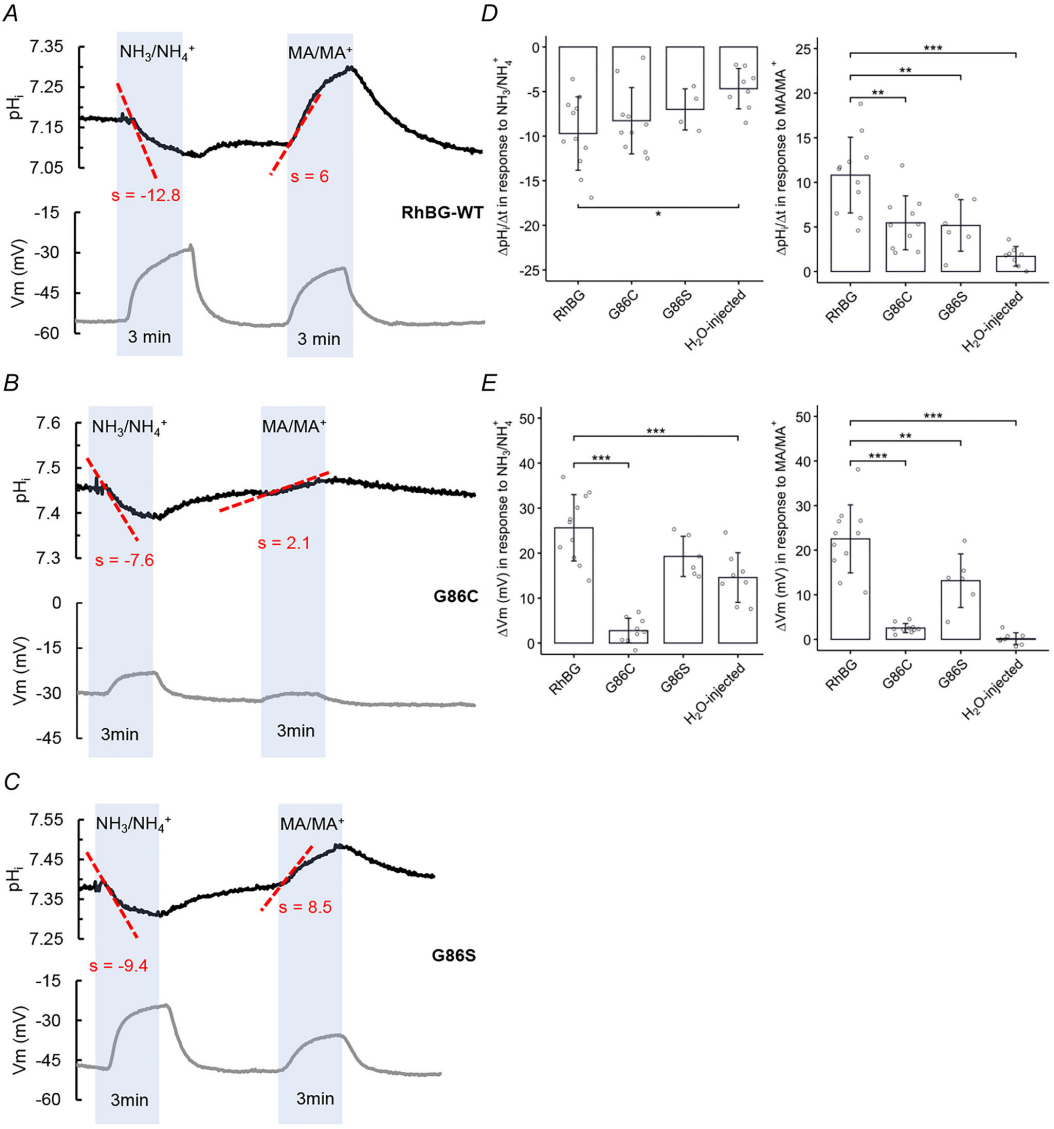
Effects of NH_3_/NH_4_^+^ and MA/MA^+^ on intracellular pH (pH_i_) on oocytes expressing RhBG mutants G86C/S pH_i_ (black tracing) and *V*_m_ (grey tracing) were measured simultaneously. s, slope ( = $\frac{\Delta \mathrm{pHi}}{\Delta \mathrm{time}(s)}$ × 10^4^). *A*, effect of NH_3_/NH_4_^+^ and MA/MA^+^ on pH_i_ in oocytes expressing human RhBG wild type (WT‐RhBG). Exposure to 5 mm NH_3_/NH_4_^+^ caused a rapid acidification (a decrease in pH_i_) with a slope of −12.8 and depolarization of the cell membrane. Exposure to MA/MA^+^ also caused cell membrane depolarization but pH_i_ alkalinization. *B*, effect of NH_3_/NH_4_^+^ and MA/MA^+^ on pH_i_ in oocytes expressing mutant RhBG_G86C_. Exposure to 5 mM NH_3_/NH_4_^+^ induced a rapid acidification (slope = −7.6) but a small depolarization of the cell membrane (Δ*V*_m_ = 6.9 mV). Exposure to MA/MA^+^ caused slight alkalinization of pH_i_ but only a limited depolarization of the cell membrane (Δ*V*_m_ = 2.7 mV) compared to the oocytes expressing WT‐RhBG. The steady state of *V*_m_ in RhBG_G86C_‐expressing oocytes is relatively low (*V*_m_ = −30 mV) compared to oocytes expressing WT‐RhBG or other RhBG mutants (*V*_m_ < −45 mV). *C*, effect of NH_3_/NH_4_^+^ and MA/MA^+^ on pH_i_ in oocytes expressing mutant RhBG_G86S_. Exposure to 5 mm NH_3_/NH_4_^+^ caused an acidification (slope = −9.4) and a depolarization of the cell membrane (Δ*V*_m_ = 24 mV), which are not different from RhBG expressing oocytes (*P* > 0.05). Exposure to MA/MA^+^ also caused a small alkalinization in pH_i_ (*P* < 0.01) and a small depolarization of the cell membrane (Δ*V*_m_ = 13.6 mV, *P* < 0.01) compared to the oocytes expressing WT‐RhBG. *D*, bar plot of the rates of change of pH_i_ (slope) in oocytes exposed to NH_3_/NH_4_^+^ (left) and MA/MA^+^ (right), respectively. *E*, bar plot of *V*_m_ changes (Δ*V*_m_) when oocytes are exposed to NH_3_/NH_4_^+^ (left) and MA/MA^+^ (right), respectively. Error bars were created using the SD. Individual data points represent individual oocyte measurements pooled from three independent injection batches after outlier removal. RhBG, *N* = 11; G86C, *N* = 10; G86S, *N* = 6; H_2_O‐injected, *N* = 8. ***P < 0.001; **P < 0.01; *P < 0.05. Statistical significance of multiple comparisons by mixed‐effects models was corrected using the false discovery rate method. For *P* values, see Table 5.

We also measured the effects of NH_3_/NH_4_^+^ and MA/MA^+^ on surface pH (pH_s_). As shown in Fig. 1*C*, NH_3_ reduced pH_s_ in RhBG‐expressing oocytes by 0.201 ± 0.053 pH units. We also found that MA induced a decrease in pH_s_ (−0.081 ± 0.030 pH units) as it did in previous Rhbg studies (Caner et al., 2015). The effects of NH_3_ entry on pH_s_ in RhBG‐expressing and H_2_O‐injected oocytes were described before at a lower NH_3_ concentration (Geyer et al., 2013). Although consistent with the results of Geyer et al. (2013), this study additionally shows the effects of MA on pH_s_ using RhBG human clone. The results of these experiments indicated similar transport patterns of human RhBG to its mouse homologue in transporting NH_3_/NH_4_^+^ and MA/MA^+^ (Abdulnour‐Nakhoul et al., 2016; Caner et al., 2015). The statistics showing significance are summarized in Figs 2*C* and *D*, 3*C* and *D* and 4*D* and *E*.

### Effects of expressing RhBG mutations on current, pH_s_ and pH_i_ changes

To evaluate the functional effects of identified variants, we measured substrate‐specific transport (NH_4_^+^, MA^+^, NH_3_ and MA) by human RhBG mutant proteins (G86C/S, G148R/W and T250A/S/M) expressed in *Xenopus* oocytes using electrophysiological methods (current, pH_s_ and pH_i_) and compared the results with WT RhBG (WT‐RhBG). ICC scores of all measurements across different groups demonstrate zero to moderate inter‐batch variation (Table 4). Table 5 summarizes the results of electrophysiological and intracellular measurements of all seven mutations compared to WT‐RhBG with adjusted *P* values of EMM contrasts.

**Table 4:** Intraclass Correlation Coefficients (ICC) for electrophysiological measurements across oocyte batches

	Measurement	Null model	Full model		Interpretation
		ICC	Unadjusted ICC	Adjusted ICC	
In response to ammonia/ammonium	Δ*I*	0.105	0.092	0.143	Minimal random effect variance for batch
	ΔpH_s_	0.189	0.152	0.278	Moderate random effect variance for batch
	ΔpH_i_/Δt	0.019	NA	NA	Random effect variance for batch is zero and ICC cannot be computed
	Δ*V*_m_	0.44	0.176	0.36	Moderate random effect variance for batch
	ΔpH_i_	0.244	0.096	0.11	Minimal random effect variance for batch
In response to methylamine/methylammonium	Δ*I*	0.129	0.015	0.055	Minimal random effect variance for batch
	ΔpH_s_	0.143	0.12	0.264	Moderate random effect variance for batch
	ΔpH_i_/Δt	0.12	0.01	0.02	Minimal random effect variance for batch
	Δ*V*_m_	0.414	0.122	0.435	Moderate random effect variance for batch
	ΔpH_i_	0.188	0.003	0.008	Minimal random effect variance for batch

ICC values were calculated to assess the consistency of measurements across batches for each parameter. The null model includes only the random effect of batch, whereas the full model includes both fixed and random effects. Unadjusted and adjusted ICC values are reported where applicable. Parameters with ICC < 0.2 were interpreted as showing minimal random effect variance for batch, whereas those with ICC between 0.2 and 0.5 were interpreted as moderate. These results support the use of pooled individual data for ANOVA and non‐parametric alternatives as the primary analysis. Abbreviations: Δ*I*, delta current; ΔpH_s_, delta surface pH; ΔpH_i_/Δt, slope; Δ*V*_m_, delta membrane potential; ΔpH_i_, delta intracellular pH.

**Table 5:** Summary table of electrophysiological measurements in oocytes compared to WT‐RhBG

	NH_4_^+^			NH_3_	MA^+^		MA	
	ΔI	slope	Δ*V*_m_	ΔpH_s_	Δ*I*	Δ*V*_m_	ΔpH_s_	slope
G86S	–	–	↓	↓	–	↓	↓	↓
*P* value	0.556	0.283	0.0167	<0.0001	0.0833	<0.0001	<0.0001	0.00220
G86C	↓	–	↓	↓	–	↓	↓	↓
*P* value	0.000200	0.437	<0.0001	0.000700	0.658	<0.0001	0.00780	0.000900
G148R	↓	–	↓	↓	↓	↓	↓	↓
*P* value	0.000100	0.0990	<0.0001	<0.0001	<0.0001	<0.0001	<0.0001	<0.0001
G148W	↓	–	↓	↓	↓	↓	↓	↓
*P* value	0.000100	0.218	0.0006	<0.0001	<0.0001	<0.0001	<0.0001	<0.0001
T250A	–	–	↓	↓	–	↓	–	–
*P* value	0.616	0.283	0.0434	0.00960	0.894	0.0187	0.439	0.387
T250S	–	–	↓	↓	–	↓	–	↓
*P* value	0.227	0.156	0.001	0.00410	0.0844	<0.0001	0.106	0.00160
T250M	↓	↓	↓	↓	↓	↓	↓	↓
*P* value	0.000300	0.0280	0.000200	<0.0001	<0.0001	<0.0001	<0.0001	<0.0001
H_2_O	↓	↓	↓	↓	↓	↓	↓	↓
*P* value	<0.0001	0.00150	<0.0001	<0.0001	<0.0001	<0.0001	<0.0001	<0.0001

Electrophysiological measurements are listed based on exposure of oocytes to either NH_3_/NH_4_^+^ or MA/MA^+^, with each measurement representing the transport of a specific solute (NH_3_, NH_4_^+^, MA or MA^+^). *P* values are generated from estimated marginal means by comparing mutants and water‐injected control with WT‐RhBG. *P* values are corrected for multiple comparisons using the false discovery rate method. –, no effect on functions; ↓: significantly impaired compared to WT‐RhBG.

#### G86C and G86S

##### Effects of G86C and G86S mutations on electrogenic NH_4_^+^ and MA^+^ transport: measured by current

Figure 2 shows NH_4_^+^ (and MA^+^)‐induced currents in oocytes expressing G86C and G86S mutants compared to WT‐RhBG. As shown in Fig. 2*A* (tracings in the light blue area), the NH_4_^+^‐induced ΔI in RhBG_G86C_‐expressing oocytes (−17.8 ± 10.4 nA, *N* = 10) was significantly smaller (*P* < 0.001) than in oocytes expressing WT‐RhBG (−35.7 ± 13.3 nA, *N* = 21). On the other hand, the NH_4_^+^‐induced current in oocytes expressing RhBG_G86S_ (−30.8 ± 10.4 nA, *N* = 10, *P* = 0.556) was not significantly different compared to that in WT‐RhBG. As shown in Fig. 2*B*, the exposure to MA/MA^+^ solution also caused an inward MA^+^‐induced current in RhBG_G86C_‐expressing oocytes (−28.6 ± 16.6 nA, *N* = 10, *P* = 0.658) and in RhBG_G86S_‐expressing oocytes (−21.2 ± 7.55 nA, *N* = 10, *P* = 0.0833) which were not significantly different from the ΔI in WT‐RhBG‐expressing oocytes (−27.1 ± 9.88 nA, *N* = 21). Figure 2*C* and *D* summarizes these data. These results showed that G86C inhibited the electrogenic transport of NH_4_^+^, whereas G86S did not affect this ability (Table 5). The findings above suggest that the transport of NH_4_^+^ and MA^+^ in *Xenopus* oocytes mediated by RhBG is probably through different mechanisms and amino acid G86 plays a critical role in this difference. A notable finding is that RhBG_G86C_‐expressing oocytes tend to have a lower *V*_m_ than oocytes expressing other RhBG mutations or WT (data not shown) even after adjusting for batch effect. This suggests a unique impact of the G86C mutation on cell membrane ionic permeability.

##### Effects of G86C and G86S mutations on NH_3_ and MA transport: measured by pH_s_

Exposing oocytes expressing WT‐RhBG to NH_3_/NH_4_^+^ solution caused rapid acidification of surface pH (pH_s_), followed by a slow recovery (Fig. 3*A*, tracings in the light blue shading). The acidification of pH_s_ is probably caused by the influx of NH_3_ into the cell. At the surface of the oocyte, influx of NH_3 s_hifts the equilibrium balance of H^+^ + NH_3_ ⇌ NH_4_^+^ to the left, leading to increased H^+^ at the surface of the membrane and hence a decrease in pH_s_. Partial recovery of pH_s_ is probably a result of subsequent slower influx of NH_4_^+^ that causes some recovery of pH_s_ (equilibrium is shifted to the right). pH_s_ of RhBG_G86C_‐ and RhBG_G86S_‐expressing oocytes decreased by 0.147 ± 0.0683 (*N* = 10, *P* < 0.001) and 0.128 ± 0.0388 pH units (*N* = 10, *P* < 0.001), respectively (Fig. 3*C*). The maximal NH_3_‐induced pH_s_ changes of these two mutations were significantly smaller than in RhBG‐expressing oocytes (−0.201 ± 0.0533 pH units, *N* = 29).

Oocytes exposed to MA/MA^+^ solution also showed a similar pattern of changes in pH_s_; acidification at the cell surface followed by small recovery (Fig. 3*B*, tracings in the light blue area). The acidification of pH_s_ is presumably caused by influx of MA, and the recovery caused by subsequent entry of MA^+^ (Abdulnour‐Nakhoul et al., 2016; Geyer et al., 2013). The slow recovery throughout the course of exposure was more prominent in oocytes expressing WT‐RhBG compared to mutations (Fig. 3*B*). The pH_s_ change induced by MA in both RhBG_G86S_‐expressing oocytes (−0.046 ± 0.015 pH units, *N* = 10, *P* < 0.001) and RhBG_G86C_‐expressing oocytes (−0.058 ± 0.029 pH units, *N* = 10, *P* = 0.00780) was significantly smaller than that in RhBG‐expressing oocytes (−0.081 ± 0.030 pH units, *N* = 29) (Fig. 3*D*). This indicates that both mutants impair the ability to transport MA in oocytes. The results above suggest that amino acid G86 plays a critical role in the transport of NH_3_ and MA in *Xenopus* oocytes mediated by RhBG.

##### Effects of G86C and G86S mutations on intracellular pH changes when exposed to NH_3_/NH_4_^+^ and MA/MA^+^

Figure 4 shows representative tracings of pH_i_ change when exposed to solutions containing either NH_3_/NH_4_^+^ or MA/MA^+^ in oocytes expressing RhBG or its mutants G86C and G86S. Exposing the RhBG‐expressing oocytes to 5 mM NH_3_/NH_4_^+^ caused a rapid decrease in pH_i_ (slope = −9.71 ± 4.12, *N* = 11) and depolarization of the cell membrane (Δ*V*_m_ = 25.6 ± 7.37 mV) (Fig. 4*A*, blue area on the left). Those changes were reversible upon removal of NH_3_/NH_4_^+^. Exposing the RhBG‐expressing oocytes to 5 mM MA/MA^+^ also resulted in depolarization of the cell membrane but caused an increase in pH_i_ (Fig. 4*A*, blue area on the right). In oocytes expressing RhBG_G86C_ (Fig. 4*B*), exposure to 5 mM NH_3_/NH_4_^+^ caused a decrease in pH_i_ (slope = −8.27 ± 3.72, *N* = 10) but a smaller depolarization of the cell membrane (Δ*V*_m_ = 2.78 ± 2.73 mV, *N* = 10, *P* < 0.001) compared to RhBG‐expressing oocytes. As described in previous studies using mouse Rhbg (Abdulnour‐Nakhoul et al., 2016; Nakhoul & Lee Hamm, 2013; Nakhoul et al., 2005), the pH_i_ decrease was induced by the entry of NH_4_^+^, which releases H^+^ in the cytoplasm. The cell depolarization was induced by the net influx of positively charged ions which in this case is attributed to the entry of NH_4_^+^. The slopes and ΔpH_i_ (Table A1) in RhBG_G86C_‐ and RhBG‐expressing oocytes exposed to NH_3_/NH_4_^+^ were not statistically significant (*P* = 0.437 and *P* = 0.622, respectively) but the Δ*V*_m_ results were (*P* < 0.001).

In RhBG_G86C_‐expressing oocytes, exposure to 5 mM MA/MA^+^ caused a smaller (ΔpH_i_ = 0.079 ± 0.045 pH units, *N* = 10, *P* < 0.001) and slower increase in pH_i_ (slope = 5.46 ± 3.03, *N* = 10, *P* < 0.001) but only a small depolarization, compared to WT‐RhBG. The increase in pH_i_ upon exposure to MA/MA^+^ is probably a result of the influx of MA across the cell membrane (Abdulnour‐Nakhoul et al., 2016) as discussed earlier.

In oocytes expressing the other mutant of the same amino acid residue, RhBG_G86S_, NH_3_/NH_4_^+^ caused pH_i_ decrease but not significantly different (*P* = 0.283) compared to oocytes expressing WT‐RhBG, whereas the depolarization induced by NH_3_/NH_4_^+^ was significantly smaller (*P* = 0.0167) (Fig. 4*C*). MA/MA^+^ caused a smaller (ΔpH_i_ = 0.064 ± 0.035 pH units, *N* = 6, *P* < 0.001) and slower increase in pH_i_ (slope = 5.17 ± 2.9, *N* = 6, *P* < 0.001) compared to WT‐RhBG. This slow increase in pH_i_ was attributed to the impaired MA transport shown in the pH_s_ results. Exposure to MA/MA^+^ also caused depolarization in RhBG_G86S_‐expressing oocytes but to a lesser extent (Δ*V*_m_ = 13.2 ± 6.01 mV, *N* = 6, *P* = 0.0167) compared to oocytes expressing WT‐RhBG (Δ*V*_m_ = 22.5 ± 7.64 mV, *N* = 11). Changes in pH_i_ and *V*_m_ in oocytes expressing mutants were also reversible upon removal of the testing solution. These data are summarized in Fig. 4*D* and *E* and Table 5.

#### G148R and G148W

##### Effects on current

**Figure 5:**
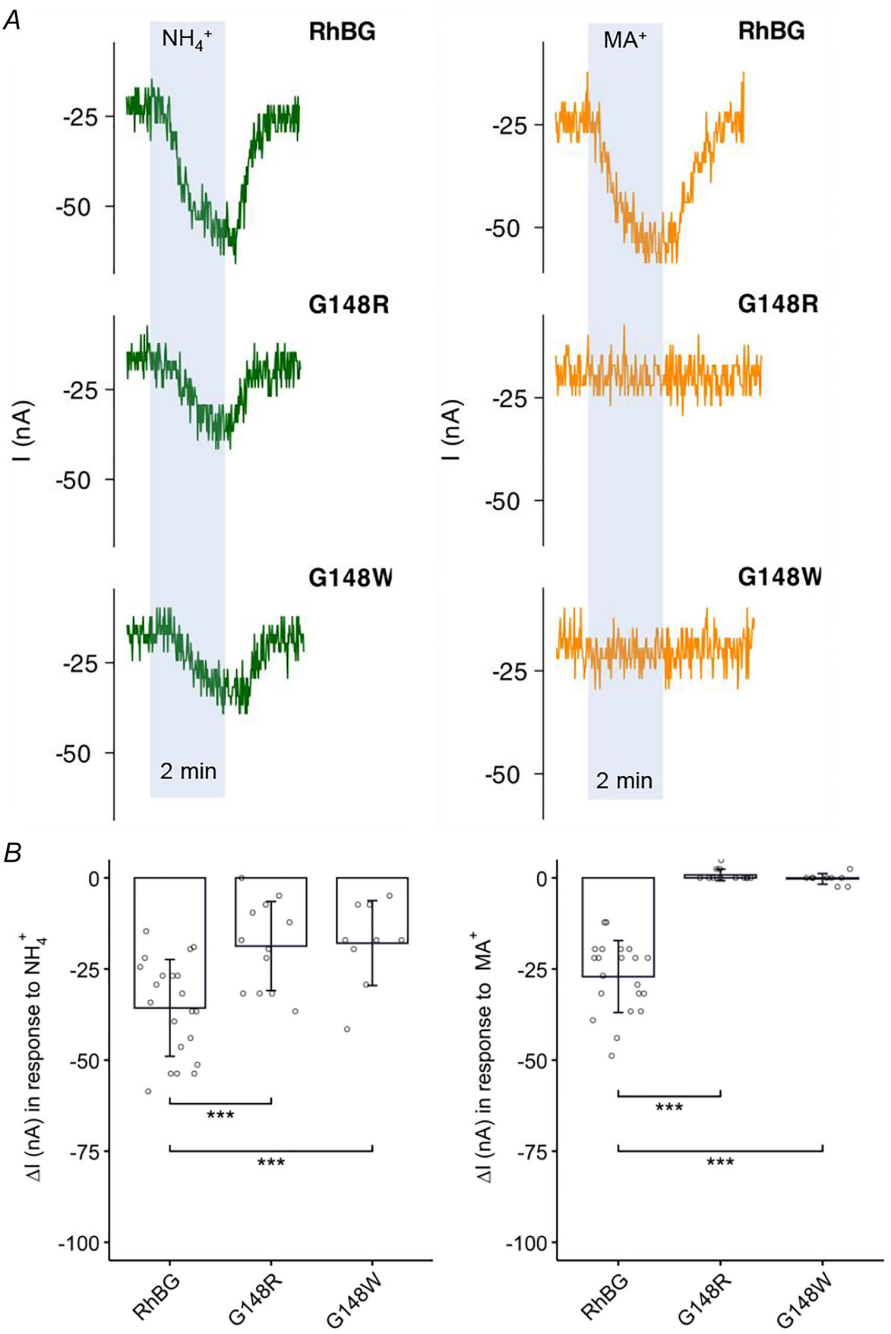
Effects of G148W/R on current induced by NH_4_^+^ and MA^+^ *A*, tracings of NH_4_^+^‐ (green) and MA^+^‐induced (orange) currents (I) of G148W and G148R mutations compared to WT‐RhBG. *B*, bar plot of current changes (Δ*I*) induced by NH_4_^+^ (left) and MA^+^ (right), in WT‐RhBG and G148W/R mutants respectively. Error bars were created using the SD. Individual data points represent individual oocyte measurements pooled from three independent injection batches after outlier removal. RhBG, *N* = 21; G148W, *N* = 9; G148R, *N* = 12. ***P < 0.001. Statistical significance of multiple comparisons by mixed‐effects models was corrected using the false discovery rate method. For *P* values, see Table 5.

NH_4_^+^‐induced inward current (Fig. 5*A*, tracings on the left) was significantly smaller in RhBG_G148R_‐ (−18.7 ± 12.2 nA, *N* = 12, *P* < 0.001) and RhBG_G148W_‐expressing oocytes (−17.9 ± 11.6 nA, *N* = 9, *P* < 0.001) compared to NH_4_^+^ currents in oocytes expressing WT‐RhBG. In oocytes expressing these two mutations, no current was recorded when exposing the oocytes to MA/MA^+^ solution (Fig. 5*A*, tracings on the right). These results indicated impaired electrogenic transport of NH_4_^+^ and MA^+^ in RhBG_G148R_‐ and RhBG_G148W_‐expressing oocytes. These data are summarized in Fig. 5*B* and Table 5.

##### Effects on pH_s_

**Figure 6:**
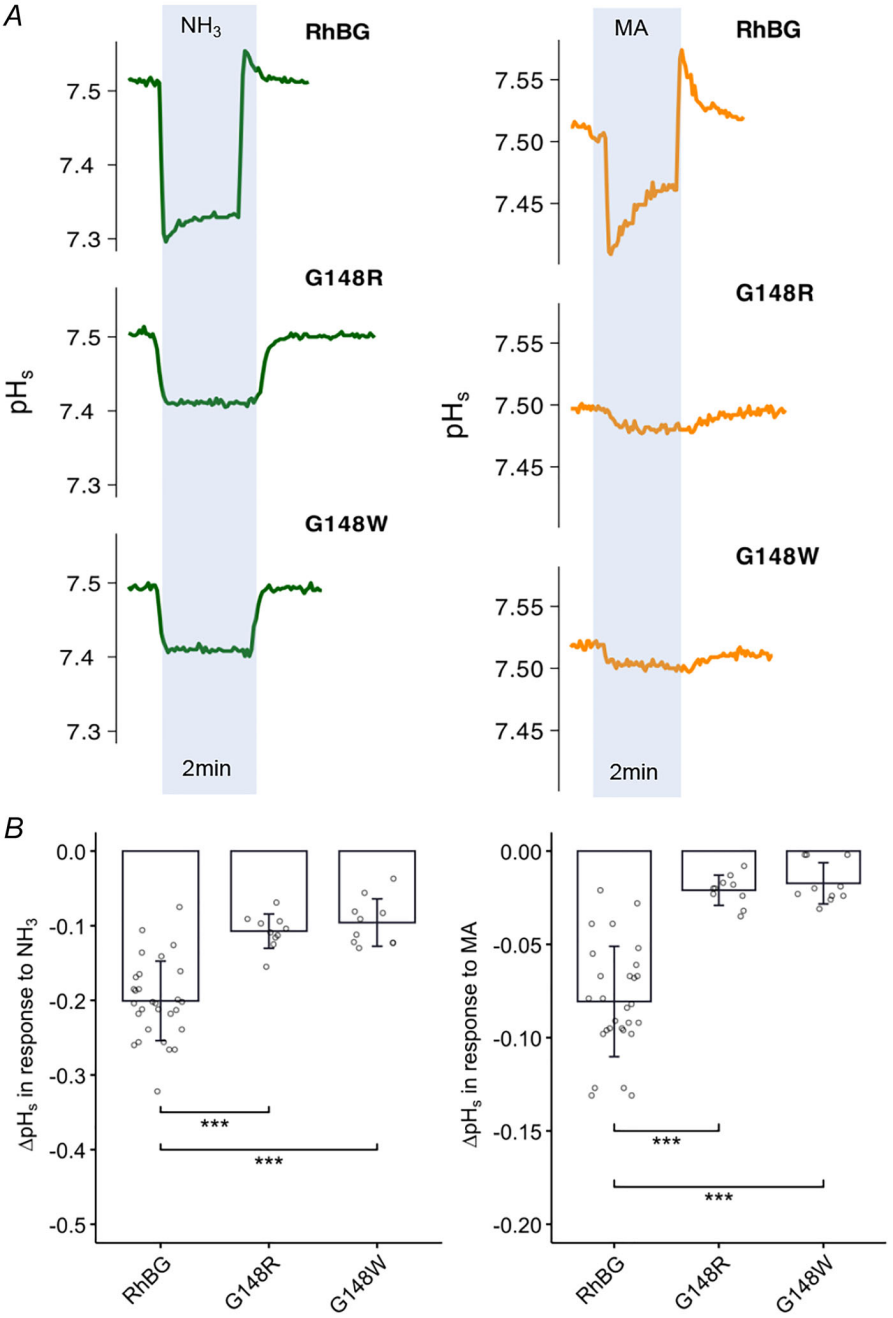
Effects of G148W/R on pH_s_ induced by NH_3_ and MA *A*, tracings of NH_3_‐induced (green) and MA‐induced (orange) pH_s_ changes of G148W and G148R mutations compared to WT‐RhBG. *B*, bar plot of pH_s_ changes (ΔpH_s_) induced by NH_3_ (left) and MA (right), respectively. Error bars were created using the SD. Individual data points represent individual oocyte measurements pooled from three independent injection batches after outlier removal. RhBG, *N* = 29; G148W, *N* = 10; G148R, *N* = 10. ***P < 0.001. Statistical significance of multiple comparisons by mixed‐effects models was corrected using the false discovery rate method. For *P* values, see Table 5.

Surface pH measurements (pH_s_) were used to investigate the effect of NH_3_ and MA on oocytes expressing the two mutations RhBG_G148R_ and RhBG_G148W._ As shown in Fig. 6*A* (left tracings in blue area), exposing WT‐RhBG oocytes to NH_3_ caused the usual fast decrease of pH_s_ followed by partial recovery. In oocytes expressing G148R, exposure to NH_3_ caused a smaller decrease of pH_s_ and there was no spontaneous pH_s_ recovery. Similar inhibition was also observed in oocytes expressing G148W. The absence of spontaneous pH_s_ recovery indicates absence of significant NH_4_^+^ transport, which is consistent with the data from current measurements as shown in Fig. 5.

Similarly, exposing oocytes to MA (right tracings) caused a typical decrease in pH_s_ followed by partial recovery in WT‐RhBG oocytes, and almost complete inhibition in G148R or G148W oocytes. These data indicate that mutations G148R and G148W completely inhibited the function of RhBG in transporting NH_3_, NH_4_^+^, MA and MA^+^. These data are summarized in Fig. 6*B* and Table 5.

##### Effects on pH_i_

**Figure 7:**
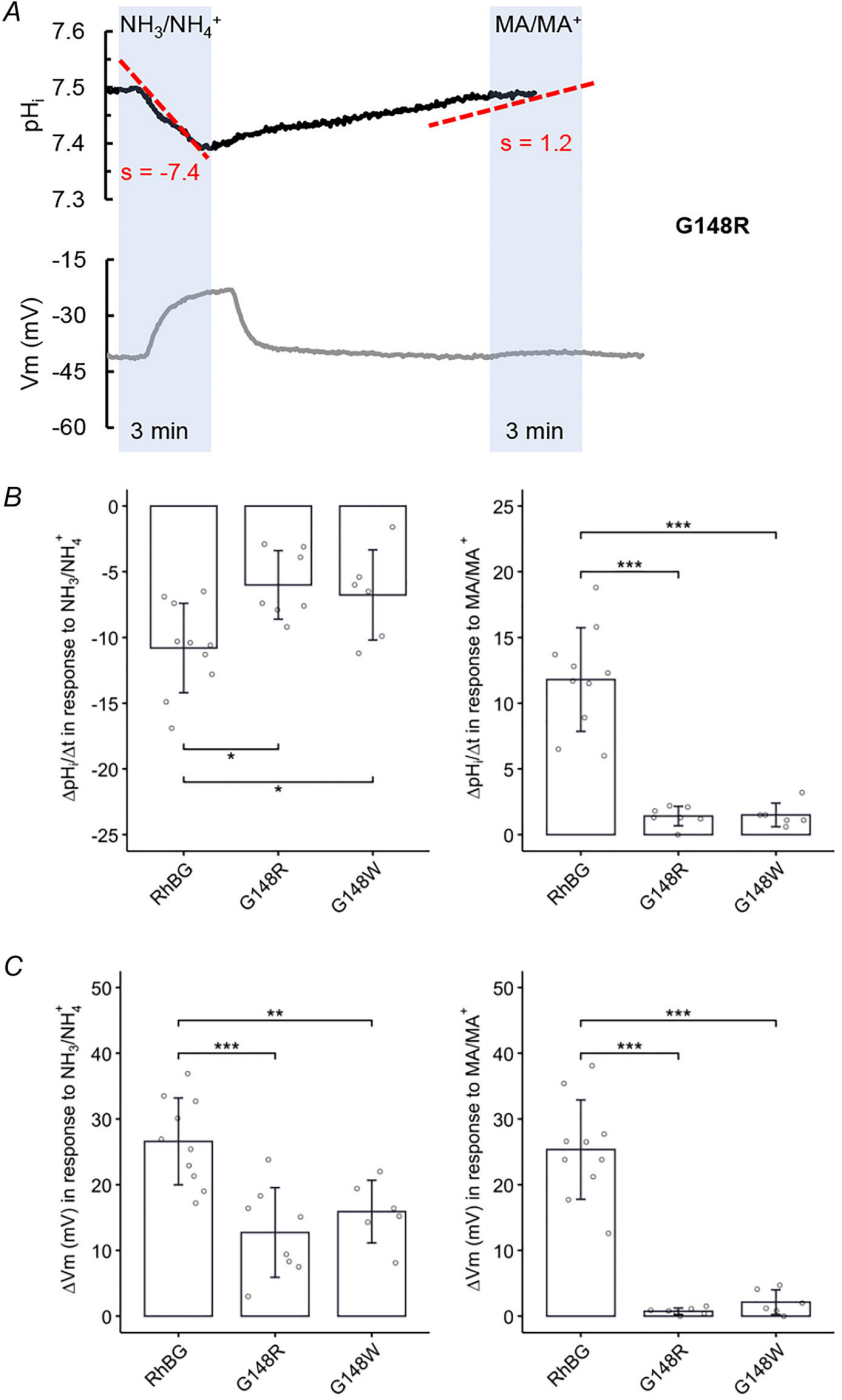
Effect of NH_3_/NH_4_^+^ and MA/MA^+^ on pH_i_ in oocytes expressing mutant RhBG_G148R_ *A*, exposure to 5 mm NH_3_/NH_4_^+^ caused a slow acidification (*P* < 0.05) and small depolarization of the cell membrane (*P* < 0.001) compared to RhBG‐expressing oocytes. Exposure to MA/MA^+^ caused a very slow increase in pH_i_ (slope = 1.2) and no depolarization of the cell membrane (*P* < 0.001) compared to the oocytes expressing WT‐RhBG. *B*, bar plot summarizing the rates of pH_i_ change (slope) when cells are exposed to NH_3_/NH_4_^+^ (left) and MA/MA^+^ (right), respectively. *C*, bar plot of *V*_m_ changes (Δ*V*_m_) of oocytes exposed to NH_3_/NH_4_^+^ (left) and MA/MA^+^ (right), respectively. Error bars were created the using SD. Individual data points represent individual oocyte measurements pooled from three independent injection batches after outlier removal. RhBG, *N* = 10; G148W, *N* = 6; G148R, *N* = 8. ***P < 0.001; **P < 0.01; *P < 0.05. Statistical significance of multiple comparisons by mixed‐effects models was corrected using the false discovery rate method. For *P* values, see Table 5.

The effects of NH_3_/NH_4_^+^ and MA/MA^+^ on pH_i_ and *V*_m_ in RhBG_G148R_‐expressing oocytes and RhBG_G148W_‐expressing oocytes were inhibited compared to WT‐RhBG and were similar to the effects in H_2_O‐injected oocytes. As shown in Fig. 7*A*, 5 mM NH_3_/NH_4_^+^ caused smaller depolarization (Δ*V*_m_ = 12.7 ± 6.82 mV, *P* < 0.001) in RhBG_G148R_‐expressing oocytes compared to WT‐RhBG oocytes (Δ*V*_m_ = 26.6 ± 6.6 mV, *N* = 10). When exposed to MA/MA^+^, RhBG_G148R_‐expressing oocytes displayed a very slow increase in pH_i_ and almost no depolarization (*P* < 0.001). Similarly, RhBG_G148W_‐expressing oocytes also had a smaller depolarization (Δ*V*_m_ = 15.9 ± 4.77 mV, *P* < 0.001) induced by NH_3_/NH_4_^+^ and almost no effect of MA/MA^+^ (tracing not shown). NH_3_/NH_4_^+^ also caused a decrease of pH_i_ in RhBG_G148R_‐expressing oocytes (slope = −6 ± 2.61, *N* = 8, *P* = 0.0990) and RhBG_G148W_‐expressing oocytes (slope = −6.77 ± 3.43, *N* = 6, *P* = 0.218) but did not reach statistical significance compared to WT‐RhBG oocytes (slope = −10.8 ± 3.4, *N* = 10). These data are summarized in Fig. 7*B* and *C* and Table 5. These experiments indicate that these mutations inhibited NH_4_^+^, MA and MA^+^ transport.

#### T250A, T250S and T250M

##### Effects on current

**Figure 8:**
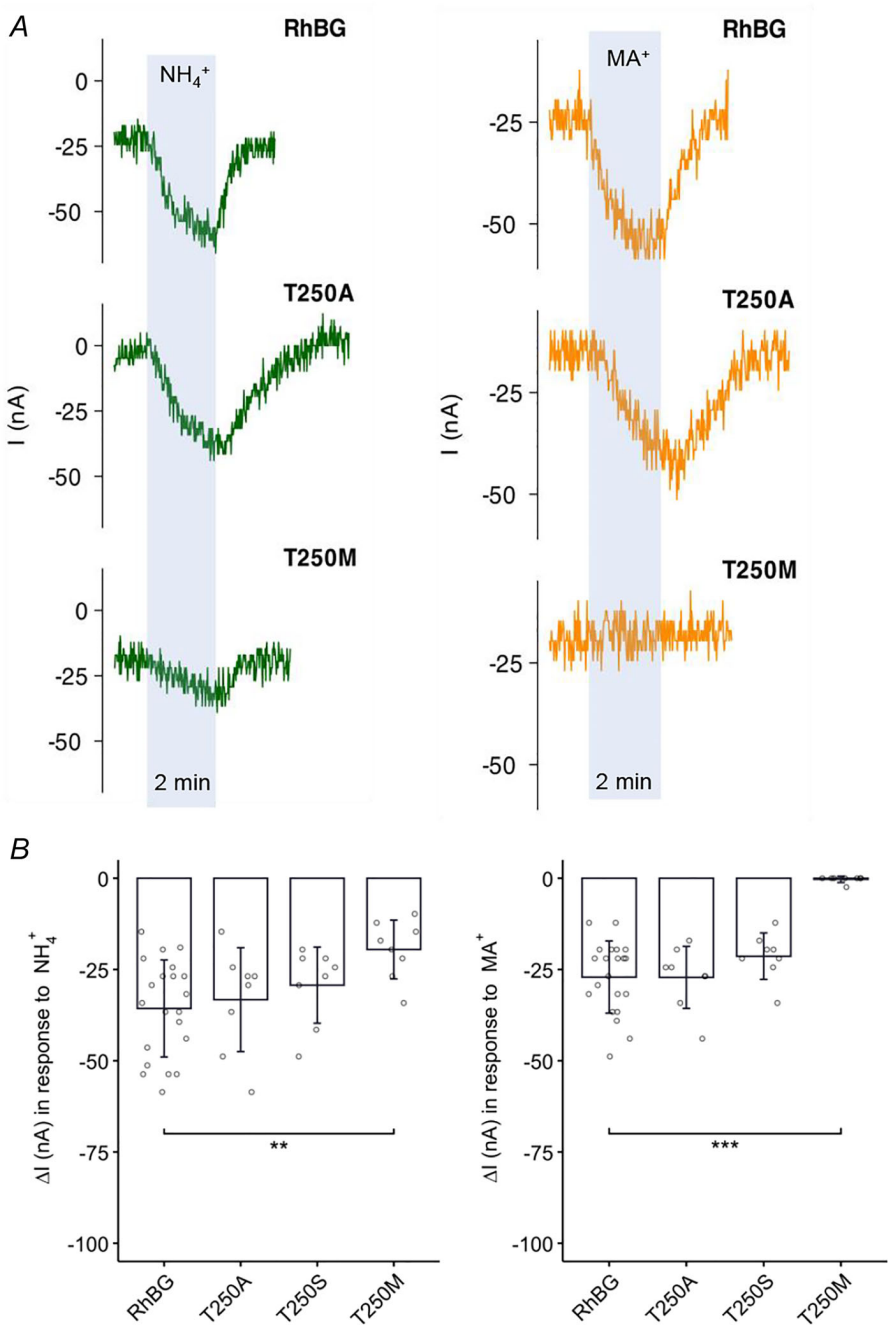
Effects of T250A/S/M on current induced by NH_4_^+^ and MA^+^ *A*, NH_4_^+^‐induced (green) and MA^+^‐induced (orange) current (*I*) in tracings for T250A and T250M mutations compared to WT. The tracing of T250S resembles T250A (tracing not shown). *B*, bar plot of current change (Δ*I*) induced by NH_4_^+^ (left) and MA^+^ (right), respectively. Error bars were created using the SD. Individual data points represent individual oocyte measurements pooled from three independent injection batches after outlier removal. RhBG, *N* = 21; T250A, *N* = 8; T250S, *N* = 8; T250M, *N* = 8. ***P < 0.001; **P < 0.01. Statistical significance of multiple comparisons by mixed‐effects models was corrected using the false discovery rate method. For *P* values, see Table 5.

T250A/S and T250M mutations of RhBG, although at the same amino acid site, are caused by different SNPs as mentioned previously (Table 3). Figure 8 shows typical experiments demonstrating the effects of these mutations on NH_3_/NH_4_^+^ and MA/MA^+^ transport. The NH_4_^+^ and MA^+^‐induced inward currents in RhBG_T250A_‐expressing oocytes (Fig. 8*A*) and in RhBG_T250S_‐expressing oocytes (tracing not shown) resembled the tracing in WT‐RhBG‐expressing oocytes with non‐significant differences in ΔI (Fig. 8*B*). RhBG_T250M_‐expressing oocytes however displayed a much smaller induced current when exposed to NH_4_^+^ (−19.5 ± 8.04 nA, *N* = 8, *P* < 0.001) compared to WT‐RhBG‐expressing oocytes (−35.7 ± 13.3 nA) (Fig. 8*B* left). No current was induced when exposing RhBG_T250M_‐expressing oocytes to MA/MA^+^ solution (Fig. 8*A*, right tracings), which was similar to H_2_O‐injected oocytes and significantly different from RhBG‐expressing oocytes (*P* < 0.001) (Fig. 8*B* right).

##### Effects on pH_s_

**Figure 9:**
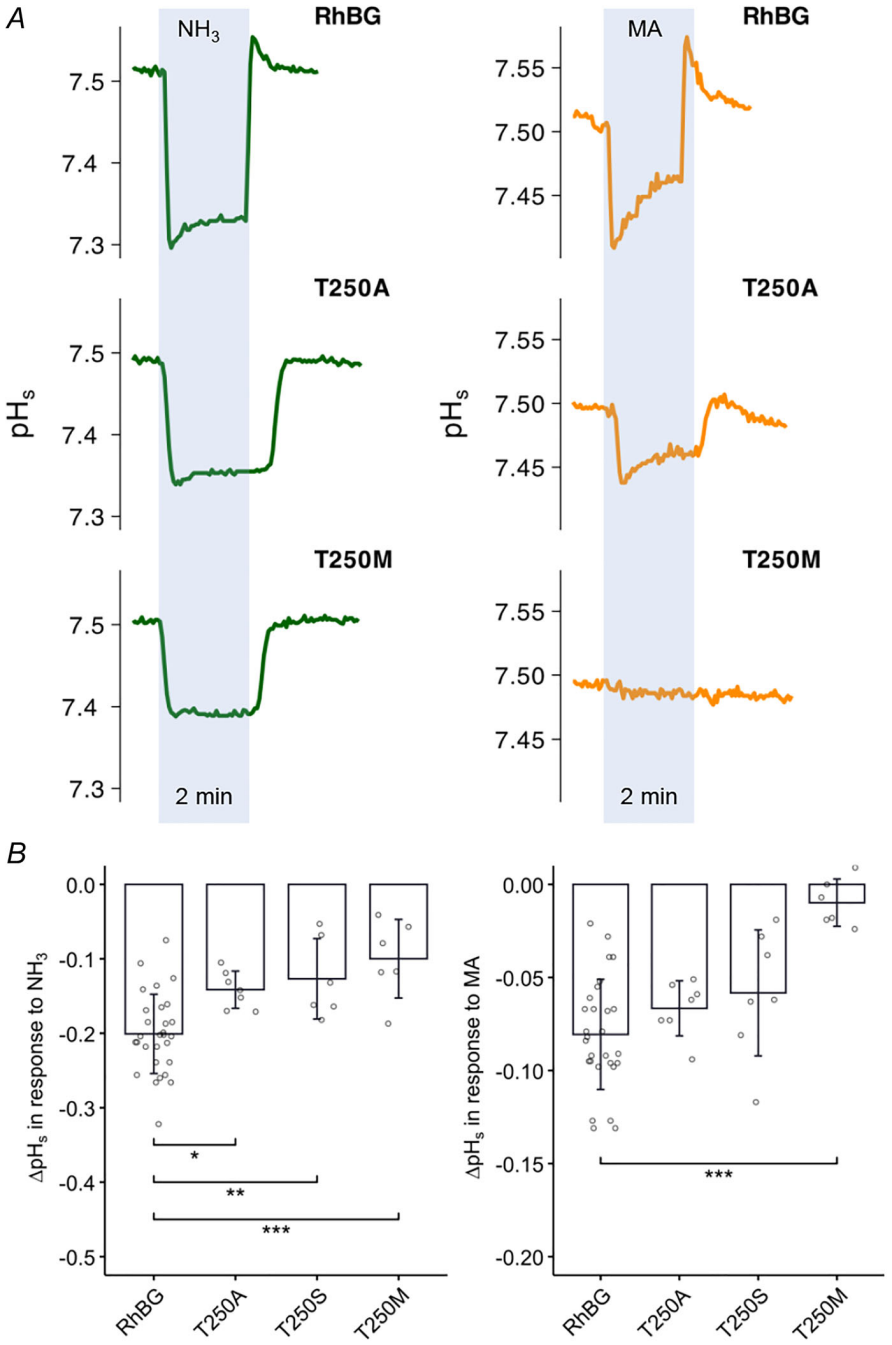
Effects of T250A/S/M on pH_s_ induced by NH_3_ and MA *A*, NH_3_‐induced (green) and MA‐induced (orange) pH_s_ changes in tracings for T250A and T250M mutations compared to WT. The tracing of T250S resembles T250A (tracing not shown). *B*, bar plot of pH_s_ changes (ΔpH_s_) induced by NH_3_ (left) and MA (right), respectively. Error bars were created using the SD. Individual data points represent individual oocyte measurements pooled from three independent injection batches after outlier removal. RhBG, *N* = 29; T250A, *N* = 7; T250S, *N* = 7; T250M, *N* = 6. ***P < 0.001; **P < 0.01; *P < 0.05. Statistical significance of multiple comparisons by mixed‐effects models was corrected using the false discovery rate method. For *P* values, see Table 5.

Figure 9 shows typical tracings of how RhBG_T250 _mutations affected NH_3_ and MA transport. As shown in Fig. 9*A* (tracings in blue area), exposing RhBG oocytes to NH_3_/NH_4_^+^ caused the usual decrease in pH_s_ followed by a partial and sustained pH_s_ recovery. In oocytes expressing RhBG_T250A_ and RhBG_T250M_, the NH_3_‐induced decreases in pH_s_ (−0.141 ± 0.025 pH units, *N* = 7, *P* = 0.00960) and (−0.127 ± 0.054 pH units, *N* = 7, *P* = 0.00410) respectively, were significantly inhibited compared to WT‐RhBG (−0.201 ± 0.053 pH units, *N* = 29). In both cases, the partial pH_s_ recovery typically observed in WT‐RhBG was absent. RhBG_T250S_ showed similar inhibition as RhBG_T250A_.

On the other hand, the MA‐induced pH_s_ changes were different between the two mutations, RhBG_T250A_ and RhBG_T250M._ As shown in Fig. 9*A* (right tracings), MA induced a typical change in pH_s_ in RhBG_T250A_, a decrease in pH_s_ followed by partial recovery as observed in WT‐RhBG. However, when RhBG_T250M_ was expressed, MA did not cause any change in pH_s_ indicating complete inhibition of MA transport. These data are summarized in Fig. 9*B* and Table 5. RhBG_T250S_ behaved similarly to RhBG_T250A._

##### Effects on pH_i_

**Figure 10:**
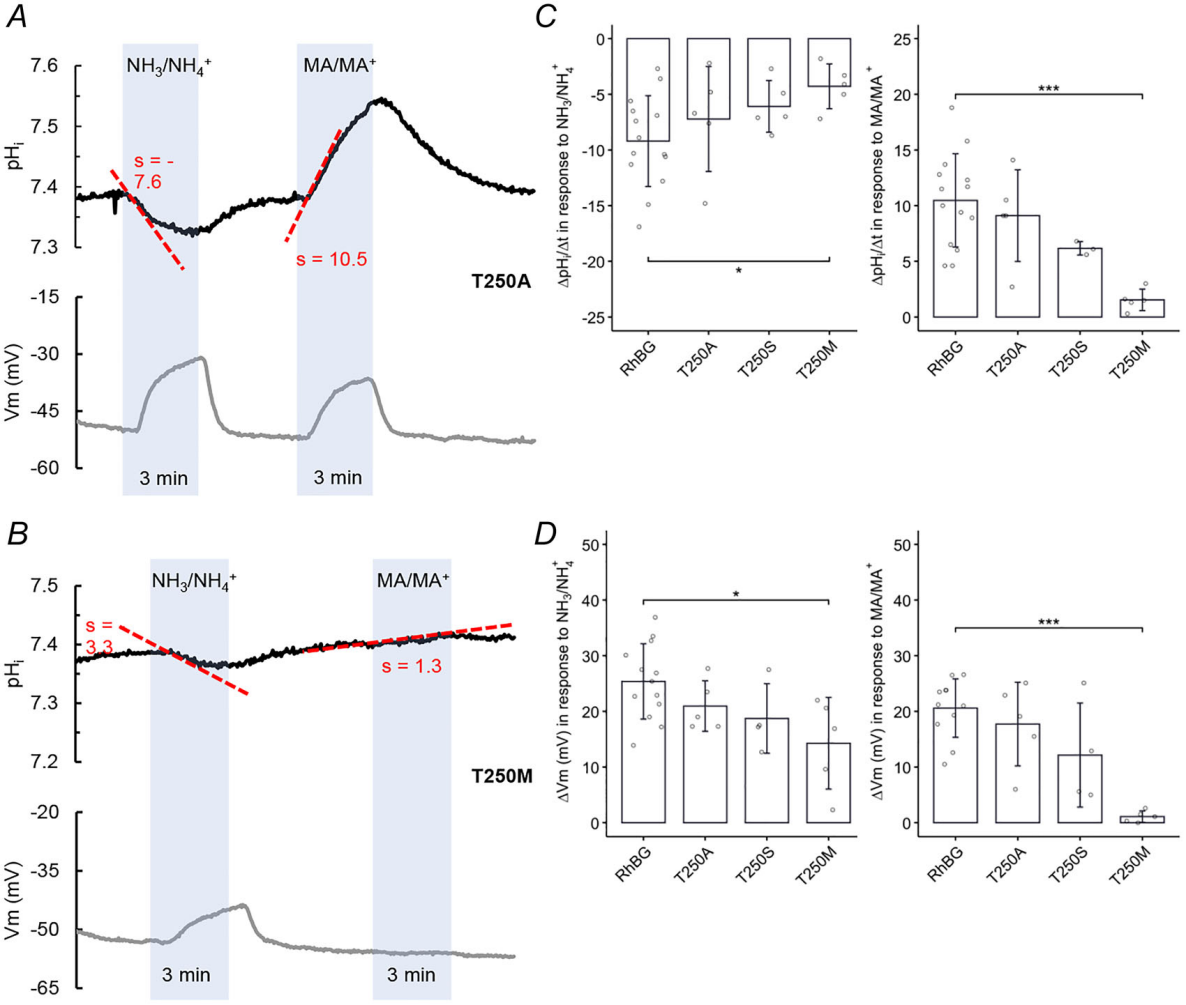
Effect of NH_3_/NH_4_^+^ and MA/MA^+^ on pH_i_ in oocytes expressing mutant RhBG_T250A_ and RhBGT_250M_ *A*, in RhBG_T250A_, exposure to 5 mm NH_3_/NH_4_^+^ caused a decrease in pH_i_ and depolarization of the cell membrane. Exposure to MA/MA^+^ caused pH alkalinization and a depolarization of the cell membrane. The results of those measurements are not different from those in oocytes expressing WT‐RhBG (*P* > 0.05). Effects of NH_3_/NH_4_^+^ and MA/MA^+^ on pH_i_ in RhBG_T250S_‐expressing oocytes (tracing not shown) showed a similar pattern to RhBG_T250A_. *B*, in oocytes expressing RhBG mutant T250M, exposure to 5 mMM NH_3_/NH_4_^+^ caused a slow acidification (*P* < 0.05) and a small depolarization of the cell membrane (*P* < 0.05) compared to oocytes expressing WT‐RhBG. Exposure to MA/MA^+^ caused a much slower increase in pH_i_ (slope = 1.3, *P* < 0.001) and no depolarization of the cell membrane (*P* < 0.001) compared to oocytes expressing WT‐RhBG. *C*, bar plot summarizing the rates of pH_i_ changes (slope) in oocytes exposed to NH_3_/NH_4_^+^ (left) and MA/MA^+^ (right), respectively. *D*, bar plot of *V*_m_ change (Δ*V*_m_) in oocytes exposed to NH_3_/NH_4_^+^ (left) and MA/MA^+^ (right), respectively. Error bars were created using the SD. Individual data points represent individual oocyte measurements pooled from three independent injection batches after outlier removal. RhBG, *N* = 14; T250A, *N* = 5; T250S, *N* = 5; T250M, *N* = 5. ***P < 0.001; *P < 0.05. Statistical significance of multiple comparisons by mixed‐effects models was corrected using the false discovery rate method. For *P* values, see Table 5.

The effects of NH_3_/NH_4_^+^ and MA/MA^+^ on pH_i_ and *V*_m_ in oocytes expressing RhBG_T250A_ (Fig. 10*A*) and RhBG_T250S_ (tracing not shown) resemble the effects in oocytes expressing WT‐RhBG (compared with Figs 1*B* and 4*A*). In five experiments, exposure to NH_3_/NH_4_^+^ caused acidification in RhBG_T250A_‐expressing oocytes with a slope of −7.22 ± 4.72 and depolarization (Δ*V*_m_ = 21 ± 4.54 mV). In RhBG_T250S_‐expressing oocytes, the average slope of pH_i_ decrease was −6.08 ± 2.32 (*N* = 5) and Δ*V*_m_ was 18.7 ± 6.25 mV. The tracings of the other mutant at the same amino acid residue site, T250M, were similar to those of H_2_O‐injected oocytes (Fig. 10*B*). In RhBG_T250M_‐expressing oocytes, exposure to 5 mM NH_3_/NH_4_^+^ caused a slow acidification (slope = −4.28 ± 2.01, *N* = 5, *P* < 0.001) and a small depolarization of the cell membrane (Δ*V*_m_ = 14.3 ± 8.24 mV, *N* = 5, *P* = 0.0125) compared to WT‐RhBG expressing oocytes (slope = −9.2 ± 4.08, Δ*V*_m_ = 25.4 ± 6.77 mV, *N* = 14). Exposure to MA/MA^+^ caused a small and slow increase in pH_i_ (slope = 1.54 ± 0.97, *N* = 5, *P* < 0.001) and no depolarization of the cell membrane (*P* < 0.001) compared to the oocytes expressing WT‐RhBG (slope = 10.5 ± 4.19, Δ*V*_m_ = 20.6 ± 5.26 mV, *N* = 14). These data are summarized in Fig. 10*C* and *D* and Table 5.

### Expression of RhBG and mutants at the oocyte membrane

**Figure 11:**
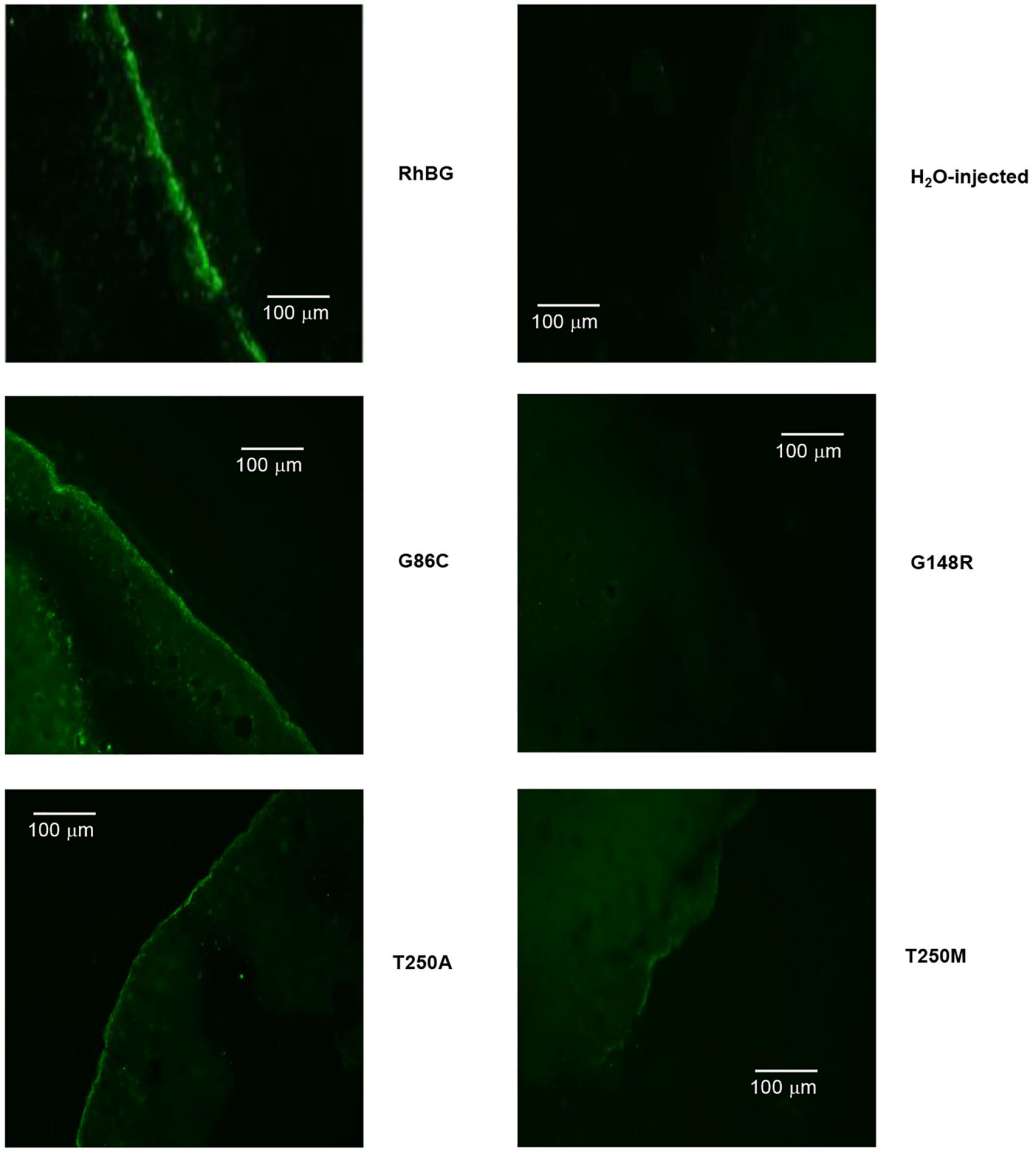
Immunohistochemistry labelling of human RhBG, G86C, G148R, T250A, T250M in oocytes RhBG, T250A and G86C‐expressing oocytes showed positive staining on the cell membrane. Labelling of T250M was weak at the cell membrane. No labelling was detected in G148R and H_2_O‐injected oocytes.

Lastly, we investigated whether the RhBG mutations affected expression of the transporters in the cell membrane. Figure 11 demonstrated immunohistochemistry (IHC) labelling of WT‐RhBG and representative mutants G86C, G148R, T250A and T250M. The IHC results of the other mutants whose transport patterns were similar to their selected pairs at the same amino acid location (G86S, G148W and T250S) were not shown. In oocytes expressing WT‐RhBG, RhBG_T250A_ and RhBG_G86C_, the cell membranes were positively and intensely stained. In oocytes expressing RhBG_T250M_, there was weak staining at the cell membrane with minimal diffusion within the cytoplasm. Oocytes expressing RhBG_G148R_ showed no staining at the cell membrane or in the cytoplasm. This expression pattern is similar to H_2_O‐injected control oocytes.

## Discussion

### CKD‐related RhBG rare variants

The results with respect to identifying rare RhBG variants related to CKD presented intriguing and important insights into the genetic underpinnings of CKD, a multifactorial disorder. Our study, utilizing WES data from the CRIC study, revealed 10 rare variants with a MAF of less than 1% in both ancestry groups. Interestingly, our findings suggest a differential distribution of these rare variants across ancestry groups, with six variants exclusively detected in individuals of European descent. Conversely, two variants were specific to the African ancestry group, whereas only one variant was present in both ancestry groups. Our analyses of association tests, employing chi‐squared and Fisher's exact tests (known for conservative type‐1 error rates, and suitable for rare variants with common disease) (Derkach et al., 2013; Lee et al., 2018; Schaid & Sinnwell, 2010), revealed four SNPs achieving statistical significance after correction for multiple comparisons. Among these, variant rs150963900 (F81L) exhibited an OR less than one, indicative of a potential protective effect against the development of CKD in European ancestry group. Multiple predictions of RhBG protein structures using machine‐learning based models (UniProt; https://www.uniprot.org) showed that F81L mutation was located at the second transmembrane domain which is also a conserved amino acid site among Rh family and across species (human, mouse and rat). Although this makes F81L a promising and interesting candidate for further investigation, the focus of the present study was on variants that had a negative effect on CKD progression and role of F81L mutation was not investigated further. Variants rs200069134, rs575652633 and rs760016272 were implicated in six distinct types of amino acid substitution (G86S, G86C, G148R, G148W, T250A and T250S). Notably, although variant rs200320178 (T250M) did not reach statistical significance, its potential impact on CKD is underscored by its occurrence at the same amino acid position as SNP rs760016272 (T250A/S), warranting further investigation. To minimize the ancestry‐related sampling bias as a result of the unequal distribution of individuals from different ancestry groups in our study population (e.g. 73% European ancestry), we conducted separate analyses for each ancestry group to assess the robustness of associations across diverse populations. Because of the small sample size of rare variants, it was not feasible to account for population stratification based on demographic factors (age, sex, etc.) within the same ancestry group. Furthermore, the inclusion of participants based on specific clinical parameters or demographic characteristics may introduce sampling bias. Future research based on more representative population cohorts should be performed to assess the robustness of the association of rare variants with CKD. Nevertheless, the identified rare variants in relation to CKD warranted closer examination of whether they affected NH_3_/NH_4_^+^ transport, an important mechanism of the role of acidosis in CKD.

### Function and expression of identified mutations

The complete functional profile of mutations and adjusted *P* values are summarized in Table 5. The functional tests described here in detail confirmed the effects of expressing the human RhBG on current, intracellular pH and surface pH changes in oocytes exposed to NH_3_/NH_4_^+^ and MA/MA^+^ solutions. Previous research (Ludewig, 2004) utilizing similar methodologies demonstrated the inward current induced by NH_4_^+^ in RhBG‐expressing oocytes but failed to observe such current with MA^+^ exposure. Notably, Ludewig (2004) only measured the current when exposed to MA^+^ at the concentration of 1 mM. In the present study, a higher concentration of MA^+^ (5 mM) did induce an inward current, as reported previously (Nakhoul, Abdulnour‐Nakhoul, Boulpaep, et al., 2010). Our study provides new insights by integrating current measurements with direct measurements of intracellular and surface pH in RhBG‐expressing oocytes exposed to NH_3_/NH_4_^+^ and MA/MA^+^. Other studies focused on the mouse homologue (Rhbg) using various methods in different expression systems (Abdulnour‐Nakhoul et al., 2016; Caner et al., 2015; Mak et al., 2006; Nakhoul et al., 2005, 2006; Nakhoul, Abdulnour‐Nakhoul, Boulpaep, et al., 2010; Westhoff & Wylie, 2006). Given that protein isoforms of the same family may play diverse physiological roles in different species or across tissues within the same species it is possible that human RhBG and mouse Rhbg can differ in functions. It was therefore important to confirm that the transport pattern of human RhBG is similar to its mouse homologue Rhbg, as our results show. This provides further biological significance and relevance for studying ammonia transporters using homologues.

Our previous studies show that Rh‐B glycoprotein transports both NH_3_ and NH_4_^+^ (Caner et al., 2015; Nakhoul et al., 2005, 2006; Nakhoul, Abdulnour‐Nakhoul, Schmidt, et al., 2010). The transport mechanisms of RhBG have been extensively studied using various experimental approaches, including radioactive uptake assays, pH measurements and stopped‐flow fluorimetry (Geyer et al., 2013; Ludewig, 2004; Mak et al., 2006; Zidi‐Yahiaoui et al., 2005). Although these studies provided valuable insights into RhBG function, the different methodological approaches have yielded varying interpretations regarding the relative contributions of electrogenic *vs*. electroneutral transport mechanisms. The technical challenges inherent in distinguishing electrogenic from electroneutral transport mechanisms in Amt/Mep/Rh proteins, including methodological inconsistencies and the complexity of multiple potential energetic pathways, have been comprehensively reviewed (Williamson et al., 2024). Although *Xenopus* oocytes contain endogenous NH_4_^+^‐permeable channels that may contribute to baseline NH_4_^+^‐induced currents, the significant differences observed between water‐injected controls and RhBG‐expressing oocytes demonstrate that the presence of RhBG contributes to the measured electrogenic responses (Fig. 1). This differential response pattern allows reliable detection of variant‐specific functional differences, which is the primary objective of this comparative study. To accurately assess these transport differences, we used a combination of measurements, as described in the Methods. Electrogenic NH_4_^+^ influx (or MA^+^) is detected as a decrease in pH_i_, an inward current, depolarization of the membrane potential (*V*_m_) and an increase in surface pH (pH_s_). On the other hand, NH_3_ influx is detected as a decrease in surface pH or as a MA‐induced increase in intracellular pH (pH_i_). Consistent with previous findings (Nakhoul, Abdulnour‐Nakhoul, Boulpaep, et al., 2010), NH_4_^+^ induced intracellular acidification, whereas MA^+^ caused initial alkalinization, reflecting the distinct transport mechanisms for these substrates. Our findings show that there are subtle differences in how the identified mutations affect either NH_3_ or NH_4_^+^ and their surrogate substrates MA and MA^+^. This complexity revealed by our study suggests that RhBG transport properties may be more nuanced than previously recognized, with different substrates potentially utilizing different transport properties.

The investigation into the effects of RhBG mutations provided valuable information about their functional roles. The findings revealed distinct and interesting impact patterns of mutations G86C and G86S on NH_3_/NH_4_^+^ and MA/MA^+^ transport. Both G86C and G86S affected NH_3_ and MA transport as evidenced by smaller ΔpH_s_ than WT‐RhBG. However, G86C appeared to be more detrimental than G86S based on the results of other measurements (effects on current and membrane potential). G86S retained the ability to transport NH_4_^+^ and MA^+^ but not the uncharged form NH_3_ and MA. It is intriguing that the substitution of glycine (G) by serine (S) appeared to disrupt the function of RhBG as a gas channel. It is possible that the hydroxyl group in serine makes it more hydrophilic than glycine, thus leading to an altered gas binding affinity or stabilization of the molecule in the hydrophobic pore of the transporter. The impact of G86C appeared to be more complex. The unique thiol group (‐SH) in cysteine (C) makes the residue not only more polar, resulting in gas affinity change, but also highly reactive. Another possibility is that cysteine is known to contribute to many protein‐protein interactions that are important for the structural integrity of proteins (Fra et al., 2017; Gutierrez et al., 2018; Jakob et al., 2023; Shi et al., 2021). This change most probably affects the RhBG structure and stability given that residue 86 is a site where the transmembrane domain turns into cytoplasmic loop (UniProt). This could also potentially explain the constantly low membrane potential in RhBG_G86C_‐expressing oocytes (Fig. 4*B*). In the pH_i_ experiments of G86C the membrane potential (*V*_m_) changes were atypically small upon exposure to NH_4_^+^ and MA^+^. One possible explanation is that, as a result of altered membrane permeability (low *V*_m_), the extent of depolarization was limited in response to NH_4_^+^ and MA^+^ in RhBG_G86C_‐expressing oocytes, which led to a significantly smaller Δ*V*_m_. The Δ*V*_m_ in RhBG_G86C_‐expressing oocytes when exposed to NH_4_^+^ was even smaller than it is in H_2_O‐injected oocytes (Fig. 4*E*) (*P* < 0.001, statistics not shown), suggesting that even endogenous total ammonia transport in oocytes was affected. The possible inhibition of endogenous NH_3_/NH_4_^+^ transporters in oocytes can also explain the discrepancy in current change (Δ*I*) in response to NH_4_^+^ (reduced) and MA^+^ (not affected). Only current measurements related to NH_4_^+^ were inhibited because native oocytes do not express MA^+^ transporter as described in previous studies (Caner et al., 2015; Nakhoul, Abdulnour‐Nakhoul, Boulpaep, et al., 2010) and the results of the present study (Figs 1*A* and 2*D*). This is additional evidence of disrupted membrane integrity and protein–protein interaction.

The findings from the expression of mutations G148R and G148W in RhBG oocytes demonstrated that they are the most detrimental to RhBG functions of all the mutations tested in the present study. The immunofluorescence staining results confirmed the absence of these two mutant proteins at the cell membrane. Residue 148 is located in the middle of a cytoplasmic loop and is conserved among RhBG homologues, suggesting this region plays a crucial structural role in RhBG function.

T250A/S and T250M mutations exhibited distinct effects on NH_3_/NH_4_^+^‐induced current responses. Although RhBG_T250A_‐ and RhBG_T250S_‐expressing oocytes behaved similarly to RhBG‐expressing oocytes, RhBG_T250M_‐expressing oocytes behaved like H_2_O‐injected oocytes. T250M demonstrated an inhibitory effect on transporting all substrates tested (NH_3_, NH_4_^+^, MA and MA^+^). RhBG_T250A_ and RhBG_T250S_ mutants maintained the overall characteristic of electrogenic NH_4_^+^/MA^+^ transport, yet both mutants partially inhibited the depolarization ability compared to WT‐RhBG. They impaired the transport of NH_3_ but not MA based on the measurements of surface pH. According to the 3D protein structure established by AlphaFold (UniProt), the T250 residue is located on the inner side (facing the centre of the pore) of a pore formed by 11 transmembrane helices and close to the extracellular loop. In its bacterial homologue AmtB, the residue aligned with T250 in human RhBG is located on the extracellular and inner side of a pore through which NH_3 _molecules are supposedly conducted (Callebaut et al., 2006). Substitution of threonine to alanine and serine altered NH_3_ transport. This suggested that the methyl group (–CH_3_) of threonine at residue 250 site may play a vital role in gas transport (NH_3_) but not NH_4_^+^. These findings add insights to the current understanding of the distinct mechanisms of NH_3_ and MA transport by RhBG.

### Mechanistic classification: membrane expression defects *vs*. per‐molecule activity impairments

Our combined electrophysiological and immunohistochemical analyses reveal that RhBG mutations impair transport through distinct molecular mechanisms, providing critical insights into structure–function relationships and potential therapeutic targets.

Mutations causing membrane expression defects represent one major category of functional impairment. The G148R (Fig. 11) and G148W mutations result in complete absence of membrane expression, with corresponding total loss of transport function. This suggests that the G148 residue, located in a conserved cytoplasmic loop, is critical for proper protein folding, trafficking, or membrane stability.

By contrast, several mutations demonstrate per‐molecule activity defects despite normal membrane expression. The T250A and T250S mutations maintain normal membrane localization but exhibit selective NH_3_ transport impairment, suggesting these residues are specifically involved in the NH_3_ transport pathway. The G86C and G86S mutations show similar patterns of normal membrane expression but differential transport effects. The G86C mutation demonstrates an ∼50% reduction in NH_4_^+^ transport, whereas G86S maintains normal electrogenic transport. These differential effects indicate that G86 is specifically required for optimal charge‐selective transport rather than protein membrane expression, and that different substitutions at residue G86 affect distinct aspects of the transport mechanism.

The T250M mutation represents a distinct hybrid defect. This mutation causes severely reduced membrane expression with complete functional impairment, suggesting this residue is critical for both protein membrane expression and transport activity. Unlike pure expression defects (G148R/W) where reduced expression fully accounts for functional loss, T250M demonstrates disproportionate functional impairment relative to its expression level, indicating that T250M specifically affects both membrane expression and transport function, contrasting with T250A/S, which only impairs transport activity.

### Heterozygous complexity: from homotrimeric models to heterozygous carrier

Although the mechanistic classification provides insights into individual mutation effects, there is an important consideration for translating these findings to clinical contexts involving the functional complexity of RhBG mutation in heterozygous patients. Functional Rhbg probably exists as a trimer (Conroy et al., 2005). It is valid to consider that the homotrimeric expression of RhBG mutants in the present study may not fully represent the heterotrimeric complexes present in heterozygous patients. Our findings represent the ‘worst case scenario’ for each mutation, as the functional tests were conducted on homotrimeric complexes. In heterozygous patients, the functional impact would be considerably more complex because of different inheritance patterns of the gene and different assembly patterns of WT and mutant subunits into trimeric complexes.

To fully address this complexity, two key biological factors must be considered: inheritance patterns and trimeric assembly mechanisms. Considering different inheritance patterns, the functional consequences could vary dramatically. Under a dominant‐negative inheritance pattern, mutant subunits would actively interfere with WT subunit function, potentially rendering entire trimers non‐functional even when containing normal subunits. By contrast, a co‐dominant pattern would result in proportional functional reduction based on the contribution of each subunit type, whereas haploinsufficiency would primarily affect total protein levels rather than individual trimer function. Furthermore, these inheritance patterns are not mutually exclusive and can co‐exist within the same mutation, creating complex phenotypes where haploinsufficiency, co‐dominant effects and dominant‐negative interference may simultaneously contribute to the overall functional impairment in heterozygous carriers.

Beyond inheritance patterns, the actual assembly of subunits into functional trimers introduces additional mechanistic complexity. Several assembly scenarios are possible in heterozygous carriers, which can be categorized based on expression levels and assembly mechanisms. With equal allelic expression and statistical assembly, trimers would contain varying ratios of wild‐type and mutant subunits, theoretically producing 12.5% all‐WT trimers, 75% heterotrimers and 12.5% all‐mutant trimers. However, assembly patterns may deviate from statistical distribution if mutations alter subunit stability, folding kinetics or assembly preferences. Additionally, cellular quality control mechanisms might selectively affect mutant subunits, influencing final trimeric composition. Furthermore, unequal allelic expression would alter subunit ratios, and non‐statistical assembly mechanisms could involve preferential incorporation, cooperative effects, or selective degradation processes. The inheritance and subunit assembly patterns for RhBG mutations remain poorly characterized in the current literature, and determining these mechanisms represents an important area for future investigation. Importantly, our homotrimeric functional studies provide a robust and valid characterization of individual mutation effects, establishing critical foundational data that will inform future heterozygous studies.

In summary, the present study provides genetic evidence linking RhBG variants to CKD risk using data from the established CRIC cohort. Our functional validation demonstrates that rare naturally occurring RhBG variants impair renal ammonia transport through distinct mechanisms: complete blockade (G148R/W and T250M) and selective substrate defects (T250A/S and G86C/S). These findings advance mechanistic understanding of RhBG‐mediated transport by identifying structural determinants that affect function and membrane expression. Moreover, the results suggest a molecular basis for clinical observations linking reduced ammonia excretion to CKD progression, independent of overt acidosis. Although validation of these associations requires further research using larger, representative cohorts to confirm CKD risk relationships, this integrated genetic and functional approach offers a genetic framework for patient stratification and personalized acid–base management, potentially identifying high‐risk patients before clinical acidosis develops.

## Additional information

## Competing interests

The authors declare that they have no competing interests.

## Author contributions

N.L.N. conceived and designed research. H.Z. performed experiments. H.Z. and N.L.N. analysed and interpreted data. H.Z., S.A‐N., L.L.H. and N.L.N. discussed results of the experiments. H.Z. prepared figures and drafted the manuscript. S.A‐N., L.L.H. and N.L.N. edited and revised the manuscript. H.Z., S.A‐N., L.L.H. and N.L.N. approved the final version of the manuscript submitted for publication.

## Funding

This work was supported in part by U54 GM104940 from the National Institute of General Medical Sciences of the National Institutes of Health, which funds the Louisiana Clinical and Translational Science Centre. Other sources of funding include a VA Merit grant BX‐001513 (S.A‐N.), Lavin‐Bernick grant (S.A‐N.), a Paul Teschan Research grant (N.L.N.) and a Tulane Bridge Fund Grant (N.L.N.).

## Supporting information


Peer Review History

## Data Availability

The data sets generated and/or analysed during the current study are available from the corresponding author on reasonable request. This includes all data related to the oocyte experiments and analytical code used for statistical analysis in RStudio. The phenotype and genotype data of the CRIC and ARIC data used in this manuscript are from NIDDK (National Institute of Diabetes and Digestive and Kidney Diseases) Central Repository and the BioLINCC (Biologic Specimen and Data Repository Information Coordinating Centre) and can be accessed through dbGaP (Database of Genotypes and Phenotypes). The dbGaP accession numbers are phs000280 for ARIC study and phs000524 for CRIC study.

## Methods

### Mixed‐effects model analysis

Mixed‐effects models were fitted using the lme4 package in R, with experimental groups as fixed effects and injection batch as a random effect. Model assumptions were verified through residual plots and normality tests. Complete model outputs including coefficients, standard errors, *t* values, and confidence intervals are presented in the Appendix (Table A1).

**Table A1:** Fixed effects estimates from linear mixed‐effects models of measurements in response to ammonia/ammonium

		Estimate	SE	d.f.	*t* value	*P_r_* (>|*t*|)		Adjusted *P*	
Δ*I*	(Intercept)	−35.589	2.769	27.139	−12.851	4.71 × 10^−13^	***		
	Experiment G86C	16.845	4.091	97.781	4.117	8.02 × 10^−5^	***	0.0002	***
	Experiment G86S	4.545	4.101	97.892	1.108	0.270498		0.5561	
	Experiment G148R	16.937	3.903	97.812	4.339	3.49 × 10^−5^	***	0.0001	***
	Experiment G148W	18.26	4.267	97.686	4.279	4.38 × 10^−5^	***	0.0001	***
	Experiment T250A	2.273	4.446	97.881	0.511	0.610325		0.6161	
	Experiment T250S	6.24	4.446	97.881	1.404	0.163577		0.2272	
	Experiment T250M	18.049	4.532	97.003	3.983	0.000132	***	0.0003	***
	Experiment H_2_O	20.123	3.179	94.009	6.33	8.29 × 10^−9^	***	<0.0001	***
ΔpH_s_	(Intercept)	−0.19817	0.01131	12.2913	−17.516	4.61 × 10^−10^	***		
	Experiment G86C	0.05105	0.01408	93.72974	3.625	0.000469	***	0.0007	***
	Experiment G86S	0.07065	0.01408	93.72974	5.017	2.48 × 10^−6^	***	<0.0001	***
	Experiment G148R	0.09105	0.01408	93.72974	6.466	4.50 × 10^−9^	***	<0.0001	***
	Experiment G148W	0.10255	0.01408	93.72974	7.283	1.00 × 10^−10^	***	<0.0001	***
	Experiment T250A	0.04498	0.01683	94.73779	2.673	0.008861	**	0.0096	**
	Experiment T250S	0.05385	0.01783	94.51925	3.019	0.003256	**	0.0041	**
	Experiment T250M	0.09086	0.01778	94.46998	5.111	1.67 × 10^−6^	***	<0.0001	***
	Experiment H_2_O	0.09788	0.01173	93.4398	8.347	6.18 × 10^−13^	***	<0.0001	***
Δ*V*_m_	(Intercept)	28.805	1.88	12.412	15.323	1.96 × 10^−9^	***		
	Experiment G86C	−24.851	2.689	79.724	−9.241	3.04 × 10^−14^	***	<0.0001	***
	Experiment G86S	−7.607	3.007	77.268	−2.53	0.013457	*	0.0167	*
	Experiment G148R	−15.141	2.745	79.028	−5.516	4.24 × 10^−7^	***	<0.0001	***
	Experiment G148W	−11.593	3.057	77.573	−3.792	0.000294	***	0.0006	***
	Experiment T250A	−6.749	3.248	76.749	−2.078	0.041049	*	0.0434	*
	Experiment T250S	−11.509	3.248	76.749	−3.544	0.000676	***	0.001	**
	Experiment T250M	−13.429	3.248	76.749	−4.135	9.00 × 10^−5^	***	0.0002	***
	Experiment H_2_O	−12.871	2.221	77.413	−5.795	1.40 × 10^−7^	***	<0.0001	***
ΔpH_i_	(Intercept)	−0.09809	0.008714	12.06691	−11.257	9.31 × 10^−8^	***		
	Experiment G86C	0.009949	0.015753	69.08451	0.632	0.52975		0.6217	
	Experiment G86S	0.010416	0.022285	78.89831	0.467	0.64151		0.6483	
	Experiment G148R	0.01805	0.016935	75.20293	1.066	0.2899		0.6073	
	Experiment G148W	0.023436	0.01906	77.3808	1.23	0.22259		0.6073	
	Experiment T250A	0.015628	0.020376	78.80194	0.767	0.44539		0.6087	
	Experiment T250S	0.018428	0.020376	78.80194	0.904	0.36854		0.6085	
	Experiment T250M	0.058028	0.020376	78.80194	2.848	0.00561	**	0.05	.
	Experiment H_2_O	0.036311	0.013887	78.93319	2.615	0.01069	*	0.05	.
ΔpH_i_/Δ*t*	(Intercept)	−9.35	0.6535	78	−14.308	<2 × 10^−16^	***		
	Experiment G86C	1.08	1.3392	78	0.806	0.422449		0.4368	
	Experiment G86S	2.35	1.9604	78	1.199	0.234272		0.2834	
	Experiment G148R	3.35	1.5425	78	2.172	0.032906	*	0.0988	
	Experiment G148W	2.5833	1.6446	78	1.571	0.120269		0.2177	
	Experiment T250A	2.13	1.7777	78	1.198	0.234467		0.2834	
	Experiment T250S	3.27	1.7777	78	1.839	0.069648	†	0.1558	
	Experiment T250M	5.07	1.7777	78	2.852	0.005558	**	0.0282	*
	Experiment H_2_O	4.9038	1.2158	78	4.033	0.000127	***	0.0015	**

Analysis results of electrophysiological measurements in response to ammonia/ammonium across different oocyte groups. The model included oocyte batch as a random effect to account for inter‐batch variability. The intercept represents the wild‐type RhBG, whereas experimental oocyte groups (mutants and water‐injected control) are modelled as fixed effects. Linearity of the data was verified using the Ramsey RESET test applied to the corresponding linear model. *P* values of estimated marginal means (EMMs) from mixed‐effects models were adjusted for multiple comparisons using the false discovery rate method. Significance levels: ****P* < 0.001, ***P* < 0.01, **P* < 0.05, †*P* = 0.05. Abbreviations: Δ*I*, delta current; ΔpH_s_, delta surface pH; Δ*V*_m_, delta membrane potential; ΔpH_i_, delta intracellular pH; ΔpH_i_/Δ*t*, slope; SE, standard error; d.f., degree of freedom; *P_r_* (>|*t*|): *P* value associated with the *t* value; Adjusted *P*, adjusted *P* value of EMMs.

## Univariate statistical analysis

All variables in electrophysiological measurements were compared using one‐way ANOVA followed by Dunnett's test for normally distributed data or Kruskal–Wallis *H* test followed by Dunn's test (non‐parametric ANOVA *post hoc* test). In *post hoc* tests, we set RhBG‐expressing group as the reference. *P* value correction for family‐wise error was built into those *post hoc* tests. Normality was tested by Shapiro's test. Outliers were identified using ‘above Q3 + 1.5 × IQR or below Q1 − 1.5 × IQR’ method and examined on dot plots. Summary data were reported as the mean ± SD. Each experimental group size was initially equally represented across all batches, though final group sizes may vary slightly as a result of outlier exclusion applied uniformly across all groups.

**Table A2:** Fixed effects estimates from linear mixed‐effects models of measurements in response to methylamine/methylammonium

		Estimate	SE	d.f.	*t* value	*P_r_* (>|*t*|)		Adjusted *P*	
Δ*I*	(Intercept)	−27.185	1.8234	44.3022	−14.909	<2 × 10^−16^	***		
	Experiment G86C	−1.658	2.9008	98.8876	−0.572	5.69 × 10^−1^		6.58 × 10^−1^	
	Experiment G86S	6.0183	3.0001	98.7134	2.006	0.0476	*	8.33 × 10^−2^	
	Experiment G148R	27.4428	2.8401	97.7202	9.663	6.71 × 10^−16^	***	<0.0001	***
	Experiment G148W	26.2774	3.1243	98.6418	8.411	3.21 × 10^−13^	***	<0.0001	***
	Experiment T250A	0.4442	3.2509	98.6231	0.137	0.8916		8.94 × 10^−1^	
	Experiment T250S	6.2417	3.2509	98.6231	1.92	0.0577	†	8.44 × 10^−2^	
	Experiment T250M	27.5298	3.2825	96.0575	8.387	4.27 × 10^−13^	***	<0.0001	***
	Experiment H_2_O	27.0009	2.3621	96.3505	11.431	< 2 × 10^−16^	***	<0.0001	***
ΔpH_s_	(Intercept)	−0.07903	0.005914	10.68968	−13.364	5.21 × 10^−8^	***		
	Experiment G86C	0.021134	0.007448	93.15029	2.838	0.00558	**	7.80 × 10^−3^	**
	Experiment G86S	0.033234	0.007448	93.15029	4.462	2.27 × 10^−5^	***	<0.0001	***
	Experiment G148R	0.058134	0.007448	93.15029	7.806	8.54 × 10^−12^	***	<0.0001	***
	Experiment G148W	0.061834	0.007448	93.15029	8.302	7.81 × 10^−13^	***	<0.0001	***
	Experiment T250A	0.006986	0.008895	94.52233	0.785	0.43417		4.39 × 10^−1^	
	Experiment T250S	0.015272	0.008895	94.52233	1.717	0.08926	†	1.06 × 10^−1^	
	Experiment T250M	0.065158	0.009395	94.19437	6.935	5.05 × 10^−10^	***	<0.0001	***
	Experiment H_2_O	0.053425	0.006283	93.94091	8.503	2.80 × 10^−13^	***	<0.0001	***
Δ*V*_m_	(Intercept)	25.258	1.659	12.105	15.223	2.93 × 10^−9^	***		
	Experiment G86C	−21.308	2.089	78.942	−10.202	4.53 × 10^−16^	***	<0.0001	***
	Experiment G86S	−11.412	2.42	76.211	−4.715	1.07 × 10^−5^	***	<0.0001	***
	Experiment G148R	−22.756	2.327	77.763	−9.777	3.49 × 10^−15^	***	<0.0001	***
	Experiment G148W	−21.554	2.461	76.437	−8.757	3.73 × 10^−13^	***	<0.0001	***
	Experiment T250A	−6.341	2.612	75.701	−2.427	0.0176	*	1.87 × 10^−2^	*
	Experiment T250S	−13.681	2.612	75.701	−5.237	1.42 × 10^−6^	***	<0.0001	***
	Experiment T250M	−22.961	2.612	75.701	−8.789	3.48 × 10^−13^	***	<0.0001	***
	Experiment H_2_O	−25.324	1.788	76.421	−14.164	<2 × 10^−16^	***	<0.0001	***
ΔpH_i_	(Intercept)	0.146908	0.006986	39.48307	21.028	<2 × 10^−16^	***		
	Experiment G86C	−0.06768	0.014171	72.79256	−4.776	9.02 × 10^−6^	***	<0.0001	***
	Experiment G86S	−0.08258	0.01735	78.86507	−4.76	8.64 × 10^−6^	***	<0.0001	***
	Experiment G148R	−0.12201	0.016289	77.31366	−7.49	9.50 × 10^−11^	***	<0.0001	***
	Experiment G148W	−0.11241	0.017387	75.28649	−6.465	9.05 × 10^−9^	***	<0.0001	***
	Experiment T250A	−0.02708	0.018764	78.25898	−1.443	0.153004		1.66 × 10^−1^	
	Experiment T250S	−0.07508	0.018764	78.25898	−4.001	0.000142	***	2.00 × 10^−4^	***
	Experiment T250M	−0.12608	0.018764	78.25898	−6.719	2.65 × 10^−9^	***	<0.0001	***
	Experiment H_2_O	−0.11459	0.012826	78.95226	−8.934	1.32 × 10^−13^	***	<0.0001	***
ΔpH_i_/Δ*t*	(Intercept)	10.7975	0.7075	36.7467	15.261	<2 × 10^−16^	***		
	Experiment G86C	−5.3036	1.4152	72.9261	−3.748	0.000355	***	9.00 × 10^−4^	***
	Experiment G86S	−5.6857	1.7262	79.0355	−3.294	0.001481	**	2.20 × 10^−3^	**
	Experiment G148R	−9.3318	1.6228	77.4174	−5.75	1.69 × 10^−7^	***	<0.0001	***
	Experiment G148W	−9.2182	1.7339	76.16	−5.317	1.02 × 10^−6^	***	<0.0001	****
	Experiment T250A	−1.6753	1.8678	78.6206	−0.897	0.372486		3.87 × 10^−1^	
	Experiment T250S	−6.4553	1.8678	78.6206	−3.456	0.000887	***	1.60 × 10^−3^	**
	Experiment T250M	−9.2353	1.8678	78.6206	−4.945	4.23 × 10^−6^	***	<0.0001	***
	Experiment H_2_O	−9.2508	1.276	79.1299	−7.25	2.46 × 10^−10^	***	<0.0001	***

Analysis results of electrophysiological measurements in response to methylamine/methylammonium across different oocyte groups. The model included oocyte batch as a random effect to account for inter‐batch variability. The intercept represents the wild‐type RhBG, whereas experimental oocyte groups (mutants and water‐injected control) are modelled as fixed effects. Linearity of the data was verified using the Ramsey RESET test applied to the corresponding linear model. *P* values of estimated marginal means (EMMs) from mixed‐effects models were adjusted for multiple comparisons using the false discovery rate method. Significance levels: ****P* < 0.001, ***P* < 0.01, **P* < 0.05, †*P* = 0.05. Abbreviations: Δ*I*, delta current; ΔpH_s_, delta surface pH; ΔVm, delta membrane potential; ΔpH_i_, delta intracellular pH; ΔpH_i_/Δ*t*, slope; SE, standard error; d.f., degree of freedom; *P_r_* (>|*t*|): *P* value associated with the *t* value; Adjusted *P*, adjusted *P* value of EMMs.

**Table A3:** Univariate statistical analysis

	NH_4_^+^			NH_3_	MA^+^		MA	
	Δ*I*	Slope	Δ*V*_m_	ΔpH_s_	ΔI	Δ*V*_m_	ΔpH_s_	Slope
G86S	−30.8 ± 10.4	−8.08 ± 4.81	13.2 ± 6.01	−0.128 ± 0.0388	−21.2 ± 7.6	10.8 ± 6.81	−0.0459 ± 0.0148	5.81 ± 4.81
*P* value	0.851	0.429	0.08137	0.000260	0.3114	0.00280	0.000490	0.00420
G86C	−17.8 ± 10.4	−8.27 ± 3.72	2.78 ± 2.73	−0.147 ± 0.0683	−28.6 ± 16.6	2.60 ± 0.990	−0.058 ± 0.0285	5.17 ± 2.90
*P* value	0.000470	0.684	1.90 × 10^−10^	0.0149	0.9985	5.40 × 10^−11^	0.0552	0.00150
G148R	−18.7 ± 12.2	−6 ± 2.61	12.7 ± 6.82	−0.107 ± 0.0230	0.8 ± 1.6	0.743 ± 0.493	−0.021 ± 0.0081	1.41 ± 0.740
*P* value	0.000380	0.0120	2.70 × 10^−4^	1.70 × 10^−6^	1.70 × 10^−15^	1.10 × 10^−8^	1.00 × 10^−9^	3.70 × 10^−7^
G148W	−17.9 ± 11.6	−6.77 ± 3.43	15.9 ± 4.77	−0.958 ± 0.0317	−0.3 ± 1.5	2.13 ± 1.88	−0.0173 ± 0.011	1.50 ± 0.897
*P* value	0.000860	0.0445	6.74 × 10^−3^	8.40 × 10^−8^	5.60 × 10^−13^	6.20 × 10^−8^	7.20 × 10^−11^	8.40 × 10^−7^
T250A	−33.3 ± 14.2	−7.22 ± 4.72	21 ± 4.54	−0.141 ± 0.0250	−27.2 ± 8.5	17.7 ± 7.51	−0.0666 ± 0.0148	9.10 ± 4.12
*P* value	0.998	0.654	0.500	0.02056	1	0.745	0.65969	0.840
T250S	−29.3 ± 10.4	−6.08 ± 2.32	18.7 ± 6.25	−0.127 ± 0.0540	−21.4 ± 6.4	10.4 ± 9.01	−0.0583 ± 0.0338	4.32 ± 2.57
*P* value	0.700	0.297	0.241	0.00392	0.427	0.0721	0.1423	0.195
T250M	−19.5 ± 8.04	−4.28 ± 2.01	14.3 ± 8.24	−0.0998 ± 0.0528	−0.3 ± 0.9	1.10 ± 1.03	−0.00983 ± 0.0127	1.54 ± 0.966
*P* value	0.00514	0.0488	0.0125	2.80 × 10^−5^	1.50 × 10^−12^	1.40 × 10^−5^	3.00 × 10^−9^	0.000280
H_2_O‐injected	−15.6 ± 7.70	−4.68 ± 2.26	14.6 ± 5.54	−0.105 ± 0.0364	0.1 ± 0.50	0.338 ± 0.173	−0.0238 ± 0.018	1.70 ± 1.10
*P* value	4.00 × 10^−7^	0.0111	0.000490	2.90 × 10^−9^	<2 × 10^−16^	7.60 × 10^−11^	1.50 × 10^−12^	1.80 × 10^−6^

All variables were compared using one‐way ANOVA followed by Dunnett's test for normally distributed data or Kruskal–Wallis *H* test followed by Dunn's test (non‐parametric ANOVA *post hoc* test). In *post hoc* tests, the RhBG‐expressing group was set as the reference. Abbreviations: Δ*I*, delta current; ΔpH_s_, delta surface pH; Δ*V*_m_, delta membrane potential; ΔpH_i_, delta intracellular pH; ΔpH_i_/Δ*t*, slope; *P* value, *P* values adjusted with built‐in methods in *post hoc* tests.
